# Spermidine Attenuates Neuroimmune Dysfunction in Gulf War Illness via Modulation of the Gut- Brain Axis

**DOI:** 10.1007/s12035-026-05763-6

**Published:** 2026-04-10

**Authors:** Ayushi Trivedi, Subhajit Roy, Madhura More, Dipro Bose, Punnag Saha, Ram Kumar, Subham Sarkar, Jonathan Skupsky, Ashok Tuteja, Kimberly Sullivan, Nancy Klimas, Saurabh Chatterjee

**Affiliations:** 1 Environmental Health and Disease Laboratory, Department of Environmental and Occupational Health, Joe C. Wen School of Population & Public Health, University of California, Irvine, CA 92697 USA; 2 Department of Pediatrics, University of California San Diego, La Jolla, CA 92093 USA; 3 Long Beach VA Medical Center, Long Beach, CA 90288 USA; 4 Division of Gastroenterology, School of Medicine, University of Utah, Salt Lake City, UT 84132 USA; 5 Department of Environmental Health, School of Public Health, Boston University, 715 Albany St. T4W, Boston, MA 02130 USA; 6 Institute for Neuro-Immune Medicine, Nova Southeastern University, Fort Lauderdale, FL 33314 USA; 7 Geriatric Research and Education Clinical Center, Miami VA Healthcare System, Miami, FL 33125 USA; 8 Division of Infectious Diseases, School of Medicine, University of California, Irvine, CA 92697 USA

**Keywords:** Gulf war illness, Spermidine, Gut–brain axis, Microbiome dysbiosis, Ahr/Nrf2/HO-1 signaling pathway, Intestinal epithelial cells, Microglia

## Abstract

Gulf War illness (GWI) affects nearly one-third of US veterans deployed during the 1990–1991 Gulf War (GW) and is characterized by chronic fatigue, neuroinflammation, and gut dysbiosis. Through comprehensive fecal metabolomics sequencing, our lab previously reported the depletion of beneficial metabolites including spermidine in the preclinical GWI mouse model. Spermidine is an endogenously synthesized polyamine known for its anti-inflammatory and mucosal barrier protective effects in various pathological diseases. Given its established role in mitigating intestinal inflammation and maintaining homeostasis, this study investigated the therapeutic potential of spermidine in a persistent (22 weeks) GWI mouse model, with a specific focus on gut–brain axis regulation. Our results demonstrated that spermidine effectively restored both microbial richness and diversity by selectively enriching beneficial bacterial taxa and suppressing growth of opportunistic pathogens, which are otherwise dysregulated following exposure to GW chemicals. Spermidine treatment also improved gut epithelial barrier integrity and reduced epithelial release of high-mobility group box 1 (HMGB1) into systemic circulation. Recent studies on GWI have implicated a critical role of gut-derived damage-associated molecular patterns (DAMPs), particularly HMGB1 in mediating neuroinflammation. Our findings indicate that systemic levels of HMGB1 critically influence the extent of blood–brain barrier (BBB) disruption and subsequent microglial activation. Mechanistically, spermidine activated intestinal aryl hydrocarbon receptor (AhR)/nuclear factor erythroid 2-related factor 2 (Nrf2)/heme oxygenase-1 (HO-1) signaling, which played a role in limiting intestinal HMGB1 release and suppressing downstream receptor for advanced glycation end-product (RAGE)–mediated microglial activation in the brain. In vitro results indicate spermidine promoted AhR/Nrf2 nuclear translocation which reduced LPS-induced HMGB1 release from primary intestinal epithelial cells (IECs), effects abrogated by AhR inhibition. Additionally, we observed that HMGB1 directly induces microglial activation via RAGE receptors in immortalized microglial (IMG) cell lines in a dose-dependent manner. These results demonstrate that spermidine decreases neuroinflammation by modulating gut–brain axis pathophysiology associated with GWI. Together, this study demonstrates the therapeutic role of spermidine in ameliorating systemic and neurological disturbances in GWI.

**Graphical Abstract:**

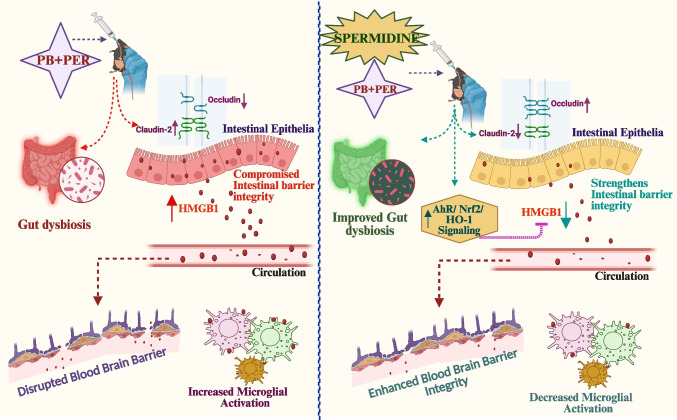

**Supplementary Information:**

The online version contains supplementary material available at 10.1007/s12035-026-05763-6.

## Introduction

An estimated 25%–32% of approximately 700,000 US veterans deployed to the 1990–1991 Persian Gulf War region developed a complex plethora of chronic symptoms like memory and cognitive impairments, headaches, fatigue, muscle pain, gastrointestinal issues, respiratory difficulties, and skin rashes, collectively referred to as Gulf War illness. To date, the pathophysiology of GWI remains complicated because of the intrinsic nature of toxic exposures which include the use of organophosphate pesticides, sarin nerve agents, pyridostigmine bromide (PB), depleted uranium, and combustion products. Furthermore, interactions with deployment-related stressors such as combat exposure, harsh living conditions, and environmental discomfort also have contributed to this multifactorial etiology [1]. Numerous epidemiological studies have reported that even decades after deployment, Gulf War veterans (GWVs) continue to exhibit a high prevalence of cardiopulmonary conditions, neurological disorders (e.g., migraines, seizures, and chronic fatigue syndrome), muscle weakness, a distinct microbiome signature, and gastrointestinal issues (e.g., irritable bowel syndrome [IBS], gut dysbiosis, and gastritis) compared to their nondeployed GW era counterparts [2–4]. Our lab recently reported a strong correlation of chronic fatigue experienced by GWV and changes in their gut microbiome structure [5]. In particular, we observed a significant increase in the *Firmicutes:Bacteroidetes* (F/B) ratio in GWV compared to nondeployed veterans associated with the degree of chronic fatigue as measured by the Multidimensional Fatigue Inventory (MFI). This microbial signature is consistent with inflammatory and microbiome patterns observed in patients suffering from myalgic encephalomyelitis/chronic fatigue syndrome (ME/CFS) [6]. Additionally, several animal studies have reported that exposure to GW chemicals, particularly PB and permethrin (PER), causes long-term alteration in gut microbiota, disrupted intestinal epithelial barrier integrity, oxidative damage, persistent neuroinflammation, and impaired neurogenesis [7–10].

HMGB1 is a highly conserved nuclear protein that functions as a potent extracellular inflammatory mediator and also as a chromatin-binding factor [11]. At basal condition, HMGB1 remains localized within the nucleus; however, in response to stress or inflammation, it translocates to the extracellular space, where it functions as a damage-associated molecular pattern (DAMP) [12]. In the context of GWI, HMGB1 has been identified as a potent proinflammatory mediator, evidenced by its increased circulating levels in both GWV and relevant GW animal models [13]. Upon its release, HMGB1 binds to pattern recognition receptors such as RAGE or Toll-like receptors (TLRs), initiating downstream inflammatory signaling cascades that contribute to renal pathology [14] as well as neuroinflammation in GWI condition [13, 15, 16]. While the exact source of this increased circulating HMGB1 in GWI remains uncertain, prior research has suggested that it may originate from the intestine, likely due to compromised intestinal barrier integrity and microbial dysbiosis [15].

GW chemical exposures have been extensively correlated with alterations of the critical gut bacteria commensals that are amenable to subsequent changes in their metabolome profiles [3, 17]. Formerly, our group explored these substantial changes in the metabolome profile analysis in a preclinical GWI mouse model [18]. In the study, spermidine, an important host-bacterial metabolite, was found to be decreased following GW chemical exposure, and numerous previous studies have long demonstrated its functional relevance across various disease conditions related to skin and cognitive function and mortality as well as inflammatory bowel disease (IBD) [19–22]. Spermidine is a natural polyamine found across various bacteria, plants, fungi, and animals ubiquitously [23]. It is synthesized endogenously through a tightly regulated process that begins with ornithine. First, ornithine is converted into putrescine by the ornithine decarboxylase (ODC1) enzyme. Next, decarboxylated S-adenosylmethionine (dcSAM) donates an aminopropyl group to putrescine via S-adenosylmethionine decarboxylase (AMD1) activity, and this reaction is catalyzed by spermidine synthase (SRM), which results in the formation of spermidine. In addition to endogenous cellular biosynthesis, spermidine levels are also influenced by two external sources: dietary intake through oral consumption and production by the intestinal microbiota [24]. Spermidine exhibits a broad spectrum of beneficial biological activities, including anti-inflammatory and antioxidant properties by influencing kynurenine pathway toward AhR/Nrf2 signaling activation [25]. Spermidine is also reported to preserve intestinal epithelial barrier integrity, activate mtROS-dependent AMPK signaling and induce Hif-1α stabilization in macrophages of IBD and colitis disease models [26, 27], and modulate microglial activation to exert neuroprotective effects in various brain diseases such as Alzheimer’s disease, traumatic brain injury, and Parkinson’s disease [28–31]. Spermidine also regulates oxidative stress [32] and promotes cellular homeostasis and longevity through its autophagy-inducing property and metabolic regulation [33, 34]. While spermidine’s independent effects on intestinal barrier integrity and neuroinflammation are well documented in various disease models, its role linking gut and brain via the gut–brain axis remains poorly defined, warranting further investigation.

While spermidine offers significant health benefits, its natural abundance in the body decreases progressively with age which might lead to impaired cellular homeostasis and vulnerability to inflammation. Chronic inflammation is a hallmark feature in GWI, which makes this age-related decrease in spermidine relevant to the GWI context. Supporting this, a metagenomics-based analysis conducted by our lab revealed a significant reduction in spermidine levels in GWI mice compared to age-matched controls within a persistent (22-week) GWI mouse model [18]. Given spermidine’s well-established anti-inflammatory and neuroprotective properties, but limited understanding of its integrative role in gut–brain axis regulation, this study was aimed to investigate its therapeutic potential in GWI condition, with a specific emphasis on its ability to modulate gut-derived systemic and neuroimmune dysfunction.

## Materials and Methods

### Animal Model and Study Design

Twelve-week-old male C57BL/6J wild-type (WT) mice were obtained from Jackson Laboratory (Bar Harbor, ME, USA). Upon arrival, the animals were housed under controlled environmental conditions, maintained at a temperature of 22–24 °C with a 12-h light/dark cycle. Mice were provided ad libitum access to standard CHOW diet and water throughout the duration of the study. The experimental design included four groups (*n* = 6 per group): control, GWI, GWI + spermidine (GWI + SPD), and SPD only. All procedures involving animals were conducted in accordance with the guidelines set forth by the National Institutes of Health (NIH) for the care and use of laboratory animals and were approved by the Institutional Animal Care and Use Committee (IACUC) at the University of California, Irvine (AUP-23-015).

### Persistent GWI Mouse Model

GW chemicals- PB and PER, along with spermidine (Cat# S0266-1G) were purchased from Sigma-Aldrich (St. Louis, MO, USA) and Millipore Sigma (Burlington, MA, USA). The control group received a vehicle solution consisting of 0.6% dimethyl sulfoxide (DMSO) in phosphate buffer saline (PBS). Mice in the GWI group were administered PB (2 mg/kg, prepared in PBS) and PER (200 mg/kg, prepared in a 0.6% DMSO with PBS) via oral gavage on consecutive days for a duration of 2 weeks. The GWI + SPD group received the same dose of GW chemicals as the GWI group. However, after 2 months of GW chemical exposure, these mice were administered spermidine (SPD) at a dose of 20 mg/kg body weight via oral gavage (the no observed adverse effect level [NOAEL] of spermidine in the mouse model is reported at 5 g/kg/BW) [35], which continued until the end of the study. A concentrated stock solution of 1 g/mL was prepared by dissolving spermidine in sterile PBS. The stock solution was subsequently diluted in sterile PBS to achieve the required working concentration for animal administration. Spermidine was fully soluble at the working concentrations used, and no visible precipitation or pH change was observed. Stock solutions were stored at 4 °C for short-term use, with fresh working dilutions prepared prior to administration. The spermidine concentration employed in our experiments was chosen in accordance with established literature report showing this dose to be biologically effective and well tolerated [36–38]. A mouse dose of 20 mg/kg corresponds to a human dose of ~1.42 mg/kg. The reported NOAEL for spermidine in humans is 13.5 mg/kg bw/day [39]. The SPD-only group received an identical SPD treatment regimen for the same duration, without prior exposure to GW chemicals. Five months post GW chemical exposure, all mice were euthanized humanely using CO_2_ asphyxiation. Blood, fecal samples, and vital organs—small intestine, brain, and liver—were carefully harvested and stored either in liquid nitrogen for molecular analysis or 10% neutral buffered formaldehyde for histopathological examination.

### Microbiome Analysis

#### DNA Extraction Methods

Fecal genomic DNA was isolated using the QIAGEN DNeasy PowerSoil Pro Kit (Cat# 47014, Qiagen, Valencia, CA, USA), according to the manufacturer’s protocol. Briefly, around 250 mg of fecal matter was added to each PowerBead Pro tube along with 800 µL of Solution CD1 and the mixture was vortexed to ensure even distribution. The tubes were then secured horizontally using a QIAGEN vortex adapter (Cat# 13000-V1-24) and vortexed at full speed for 10 min to achieve mechanical lysis of microbial cells. Following homogenization, the tubes were centrifuged at 15,000 × *g* for 1 min to pellet debris. Around 500–600-µL supernatant was transferred to a new 2-mL microcentrifuge tube. Next, 200 µL of Solution CD2 was added, and the tubes were vortexed briefly. After another centrifugation at 15,000 × *g* for 1 min, the clarified supernatant was moved to a fresh tube. Six hundred–microliter Solution CD3 was then added to the supernatant. Aliquots of 650 µL were loaded onto MB Spin Columns in two rounds, each spun at 15,000 × *g* for 1 min to ensure all lysate passed through. The flow-through was discarded after each spin. Columns were placed into clean collection tubes, and a wash step was performed with 500 µL of Solution EA, followed by another spin at 15,000 × *g* for 1 min. After discarding the flow-through, a second wash with 500 µL of Solution C5 was performed under the same conditions. A final spin at 16,000 × *g* for 2 min ensured the column was dry. DNA elution was carried out by transferring each column to a fresh 1.5-mL elution tube, and 50–100 µL of Solution C6 was applied directly to the membrane. A final spin at 15,000 × *g* for 1 min yielded purified genomic DNA, which was stored at −80 °C until further use. DNA concentration was measured using the Qubit 4 fluorometer with the Qubit™ dsDNA High Sensitivity Assay Kit (Thermo Fisher Scientific, Grand Island, NY, USA) to ensure sufficient yield and quality.

#### Library Preparation and Sequencing Methods

DNA libraries were prepared using the xGen DNA Library Prep Kit (Cat# 10009821, Integrated DNA Technologies [IDT], Iowa, USA) and xGen Normalase UDI Primers (Cat# 10009796, IDT) with a total DNA input of 1.5 ng. Genomic DNA was first fragmented enzymatically using a proportional amount of the xGen fragmentation enzyme optimized for low-input samples. Following fragmentation and end-repair, ligation of xGen UDI adapters was performed using the ligation master mix provided in the kit, ensuring each sample received a unique combination of dual indices for multiplexed sequencing. Subsequently, adapter-ligated DNA fragments were amplified using PCR for 10 cycles to construct libraries. The samples were amplified using the following thermal cycling program: 98 °C for 45 s, followed by 10 cycles of 98 °C for 15 s, 60 °C for 30 s, and 72 °C for 30 s, with a final extension at 72 °C for 5 min to complete library amplification. DNA libraries were purified using AMpure magnetic Beads (Beckman Coulter, Cat# A63881) at a 0.6× ratio. The plate was shaken at 1800 rpm for 3 min, incubated for 5 min at room temperature, and placed on a magnetic stand for 2 min to separate the beads. Beads were washed twice with 200 µL of 80% ethanol, air-dried for 10 min, and DNA was eluted in 50 µL of QIAGEN Elution Buffer (EB). Final libraries were quantified using a Qubit 4 fluorometer with the Qubit™ dsDNA HS Assay Kit. Libraries were pooled in equimolar concentrations and sequenced on an IIllumina NovaSeq X Plus platform with a paired-end 2× 150 bp read format, targeting approximately 20 million reads per sample.

Bioinformatics analysis was carried out via the CosmosID-HUB platform. The system utilizes a high performance data-mining k-mer algorithm that rapidly disambiguates millions of short sequence reads into the discrete genomes engendering the particular sequences. The pipeline has two separable comparators. The first consists of a precomputation phase for reference databases and the second is a per-sample computation. The input to the precomputation phase is databases of reference genomes, virulence markers, and antimicrobial resistance markers that are continuously curated. The output of the precomputational phase is a phylogeny tree of microbes, together with sets of variable length k-mer fingerprints (biomarkers) uniquely associated with distinct branches and leaves of the tree. The second per-sample computational phase searches the hundreds of millions of short sequence reads, or alternatively contigs from draft de novo assemblies, against the fingerprint sets. This query enables the sensitive yet highly precise detection and taxonomic classification of microbial NGS reads. The resulting statistics are analyzed to return the fine-grain taxonomic and relative abundance estimates for the microbial NGS datasets. To exclude false-positive identifications, the results are filtered using a filtering threshold derived based on internal statistical scores that are determined by analyzing a large number of diverse metagenomes. The same approach is applied to enable the sensitive and accurate detection of genetic markers for virulence and for antibiotic resistance.

Permutational multivariate analysis of variance (PERMANOVA) was carried out to investigate bacterial microbiome diversity, and alpha diversity indices (Chao1, Shannon) were calculated to evaluate within-sample diversity, while beta diversity metrics (Bray–Curtis) were used to assess variation between samples.

Entire sequencing data is available via the Sequence Read Archive (SRA) database, accession ID: PRJNA1307016: Spermidine Attenuates Neuroimmune Dysfunction in Gulf War Illness via Modulation of the Gut–Brain Axis.

### Cell Culture

#### Cell Lines

C57BL/6J primary small intestinal epithelial cells (IECs) (Cat# C57-6051) were obtained from Cell Biologics (Chicago, IL, USA), and the mouse immortalized microglial (IMG) cell line (Cat# SCC134) was purchased from Millipore Sigma (Rockville, MD, USA). rHMGB1 (Cat#764006) was obtained from BioLegend (San Diego, CA, USA). Cell culture reagents- high-glucose Dulbecco’s Modified Eagle Medium (DMEM) supplemented with pyruvate (Cat# 11995065) and 0.5% Trypsin–EDTA without phenol red (Cat# 15400054)—were both from Thermo Fisher Scientific. Penicillin–streptomycin solution (Cat# 15140-122) and L-Glutamine 200 mM (100x) (Cat# 25030-081) were acquired from Gibco, Thermo Fisher Scientific, whereas heat-inactivated fetal bovine serum (FBS) (Cat# F4135-500ML) was purchased from Sigma-Aldrich. The AhR antagonist, CH223191 (Cat# C8124-5MG), insulin–transferrin–sodium selenite (Cat# I3146-5ML) was obtained from Millipore Sigma, and lipopolysaccharide (LPS) from *Escherichia coli* O111:B4 (Cat# 19661) was acquired from Cayman Chemical (Ann Arbor, MI, USA).

#### Cell Culture Treatment

Primary IECs were cultured in DMEM supplemented with 10% FBS and 1× insulin–transferrin–selenium. Upon reaching 70% confluency, cells were serum-starved for 12–18 h and pretreated with 1 mM spermidine for 2 h [28], followed by stimulation with LPS (0.5 μg/mL). In this study, LPS was used as a standardized immune stimulus to probe inflammatory signaling in primary IECs relevant to the mechanistic pathways implicated in GWI [40, 41]. A working concentration of 0.5 µg/mL LPS was prepared by diluting a 10 µg/mL LPS stock solution into complete DMEM. The stock solution was vortexed gently before dilution to ensure homogeneity, and the final working solution was prepared fresh for each experiment. Similarly, a highly concentrated 6.9 M spermidine stock was prepared by dissolving spermidine in sterile PBS. This stock solution was subsequently diluted in complete DMEM to obtain the final 1 mM working concentration. CH223191, an AhR inhibitor, was used to assess the role of AhR signaling due to spermidine treatment. CH223191 powder was weighed and dissolved in DMSO, and a 10 mM stock solution was prepared in DMEM. The stock was vortexed until fully solubilized, and immediately before treatment, the 10 mM stock was further diluted in complete DMEM to obtain a 10 µM working solution. We also employed Nuclear/Cytosol Fractionation Kit (Cat# ab289882, Abcam) to separate nuclear and cytoplasmic protein fractions to determine AhR translocation from the cytoplasm to the nucleus. Further evaluation of the activation status of the AhR/Nrf2/HO-1 signaling was performed by western blotting, and AhR nuclear localization was validated through immunofluorescence. Lastly, HMGB1 release was quantified by ELISA in cell culture supernatants.

IMG cell lines were cultured in DMEM supplemented with 10% FBS and 1× L-Glutamine and treated with increasing concentrations of rHMGB1 (0, 10, 100, 1000, and 10,000 ng/mL). The reported ED_50_ of rHMGB1 used in this study has a range of 3–25 μg/mL. Immunofluorescence staining was used to investigate whether HMGB1 induces microglial activation through RAGE signaling via HMGB1-RAGE and RAGE-IBA1 colocalization.

### Immunofluorescence

Paraffin-embedded small intestine and brain tissue sections were deparaffinized using standard xylene and ethanol gradient procedure. Antigen retrieval was performed by incubating the slides in a commercially available antigen retrieval solution within a laboratory-grade steamer (IHC-World, Woodstock, MD, USA) for 30–35 min to unmask formalin-fixed epitopes. Tissue sections were then permeabilized in PBS containing 0.1% Triton X-100 (PBS-T). Nonspecific binding was blocked using 5% goat serum for one hour. The sections were then incubated overnight at 4 °C with primary antibodies—anti-occludin (1:300; AB216327), anti-claudin-2 (1:250; AB53032) from Abcam (Cambridge, MA, USA), anti-CD-31 (1:250; NB100-2284, Novus Biologicals, Centennial, CO, USA), anti-claudin-5 (1:250; sc-374221), anti-RAGE (1:250; sc-365154) from Santa Cruz Biotechnology (SCBT) (Dallas, TX, USA), anti-HMGB1 (1:250; 10829–1-AP), anti-IBA1 (1:250; 10904-1-AP), and anti-AhR (1:300; 28727-1-AP) from Proteintech (Rosemont, IL, USA). After primary antibody incubation, species-specific secondary antibodies conjugated to Alexa Fluor 488 or 633 (Invitrogen, Waltham, MA, USA) were applied. The sections were washed thoroughly and mounted using ProLong Gold antifade reagent containing 4′,6-diamidino-2-phenylindole (DAPI) (Life Technologies, Carlsbad, CA, USA). Imaging was performed with an Olympus BX43 microscope (Olympus, Center Valley, PA, USA), and morphometric analysis was conducted using CellSens Software version 2.2 (Olympus America).

### Immunohistochemistry

Following antigen retrieval, the sections were incubated with 3% hydrogen peroxide (H_2_O_2_) for 20 min to inhibit endogenous peroxidase activity and then washed three times with 1× PBS. Tissue sections were incubated in 5% goat serum for 1 h at room temperature and later overnight at 4 °C with primary antibodies prepared in blocking buffer (IHC-World): anti-HMGB1 (1:200; sc-135809), from SCBT, and anti-IBA1 (1:250; 10904-1-AP) from Proteintech. Secondary incubation was performed using species-specific biotinylated antibodies—anti-mouse (1:300; BA-9200) and anti-rabbit (1:300; BA-1000) obtained from Vector Laboratories (Burlingame, CA, USA) followed by incubation with a streptavidin-horseradish peroxidase (HRP) conjugate from the Vectastain Elite ABC Kit (Cat# PK-6100) at a 1:500 dilution. Immunostaining was visualized using 3,3′-diaminobenzidine (DAB, Sigma-Aldrich) and Mayer’s hematoxylin (Sigma-Aldrich) for nuclear counterstaining. The stained sections were mounted using mounting medium from Abcam. Bright-field images were acquired using an Olympus BX43 microscope, and quantitative morphometric analysis was conducted using CellSens Software v2.2.

### Quantitative Real-Time Polymerase Chain Reaction (qRT-PCR)

Total RNA was extracted using TRIzol reagent (Invitrogen) in combination with the RNeasy Mini Kit (Cat# 74104, Qiagen, Valencia, CA, USA), following the manufacturer’s instructions. For cDNA synthesis, 1 μg of RNA was used with the iScript cDNA synthesis kit (Cat# 1708891, Bio-Rad, Hercules, CA, USA). The reverse transcription protocol included a priming step at 25 °C for 5 min, followed by reverse transcription at 48 °C for 20 min, and a final inactivation step at 95 °C for 1 min. qRT-PCR was carried out using SsoAdvanced SYBR Green Supermix (Bio-Rad) on an AriaMx Real-Time PCR System (Agilent Technologies, Santa Clara, CA, USA), with gene-specific primers listed in Table 1. The cycle threshold (Ct) values for CYP1A1 were normalized to the housekeeping gene GAPDH, and relative gene expression was calculated using the 2^−ΔΔCt^ method.

**Table 1:** Mouse primer sequence

Gene	Primer sequence (5′−3′)		Melting temperature (Tm)
	Forward sequence	Reverse sequence	
GAPDH	CGACTTCAACAGCAACTCCCACTCTTCC	TGGGTGGTCCAGGGTTTCTTACTCCTT	62 °C
CYP1A1	CAATGAGTTTGGGGAGGTTACTG	CCCTTCTCAAATGTCCTGTAGTG	60 °C

### Western Blotting

Total protein was isolated using 1× RIPA lysis buffer mixed with protease and phosphatase inhibitors, and concentration was quantified using the BCA Protein Assay Kit (Thermo Fisher Scientific, Rockford, IL, USA). Approximately 30 µg of protein was mixed with 4× NuPAGE™ LDS Sample Buffer and 10% β-mercaptoethanol (Thermo Fisher Scientific, Rockford, IL, USA). Proteins were separated by SDS-PAGE using a Novex 4–12% Bis–Tris gradient gel and transferred onto nitrocellulose membranes using the Trans-Blot Turbo Transfer System (Bio-Rad, Hercules, CA, USA). After transfer, membranes were stained with Ponceau S to confirm protein loading, followed by blocking in 3% bovine serum albumin (BSA) prepared in 1× TBST for 1 h at room temperature. Membranes were incubated overnight at 4 °C with the following primary antibodies in 1% BSA in TBST: anti-AhR (1:1000; 28727-1-AP, Proteintech), anti-Nrf2 (1:800; 12721S), anti-HO-1 (1:1000; 43966S), anti-β actin (1:2500; 4970S), and anti-Histone H3 (1:1500; 4499S), sourced from Cell Signaling Technology [CST] (Danvers, MA, USA).

Species-appropriate HRP-conjugated secondary antibodies (1:3000–1:5000; CST) were then applied to detect bound primary antibodies. Pierce ECL Western Blotting Substrate (Thermo Fisher Scientific, Waltham, MA, USA) was used to develop blots, and imaging was performed on the ChemiDoc™ Imaging System (Bio-Rad, Hercules, CA, USA). Band intensities were quantified using Image Lab Software version 6.0.1 (Bio-Rad).

### ELISA

HMGB1 levels were quantified in mouse serum samples and in the culture supernatant of treated IECs using a mouse HMGB1 ELISA kit (Cat# RK06737) from ABclonal (Woburn, MA, USA).

### Statistical Analysis

Data analysis was conducted using R (version 4.5.0) and GraphPad Prism (version 10.5.0, GraphPad Software, San Diego, CA, USA). Chao1 richness and Shannon diversity indices were calculated using the fossil package, and Bray–Curtis dissimilarity was calculated using PERMANOVA through the vegan package. Statistical significance for Chao1 and Shannon diversity was determined using the Kruskal–Wallis test followed by pairwise Wilcoxon rank-sum tests as post hoc comparisons. Principal coordinate analysis (PCoA) was performed with vegan to visualize clustering patterns for operational taxonomic units (OTUs). For analysis involving four groups, statistical analyses were evaluated by one-way analysis of variance (ANOVA) followed by Tukey’s Honest Significant Difference (HSD) post hoc test via the multcomp package. Both normality and homogeneity of variances were tested using the Shapiro–Wilk normality test and Levene’s test. If homogeneity of variances was unequal, Welch’s ANOVA with Games–Howell post hoc test was used. If data did not follow normal distribution, Kruskal–Wallis test followed by Dunn’s post hoc was used. All plots and data visualizations were generated using the ggplot2 package. AhR intensity profiles and microglial branch length were quantified using FIJI software (ImageJ, version 1.54p, NIH) by generating linear regions of interest (ROIs) across the cellular axis and applying the “Skeletonize” function to analyze microglial morphology. A mechanistic network showing the association between GWI pathology and spermidine-mediated protection was constructed using R packages- igraph, ggraph, tidygraph, and ggplot2. This network integrates experimental findings into a visually interpretable flow of events, linking PB + PER exposure to downstream molecular and cellular disruptions in the gut–brain axis, and highlights how spermidine intervenes to mitigate these outcomes. The network does not involve any statistical testing and is schematic in nature; however, all relationships are supported by statistically validated experimental data presented elsewhere in the study. Results are presented as mean ± standard deviation (SD) or mean ± standard error of the mean (SEM), with individual significant *p*-values provided in the corresponding results section or figure legends. Statistical significance was defined as *p* < 0.05.

## Results

### Spermidine Modulates Microbiota Diversity and Composition in GWI-Induced Mice

**Fig. 1:**
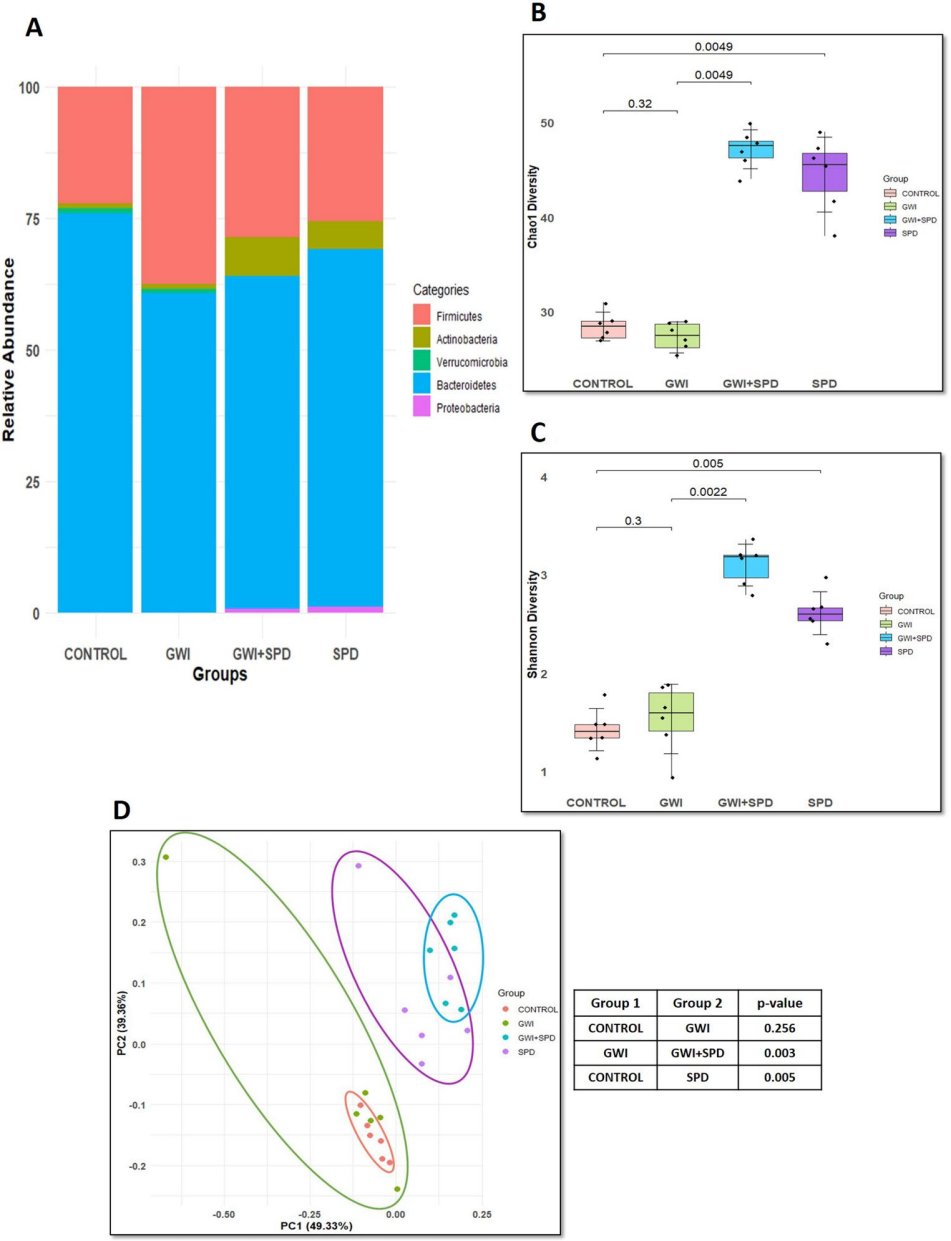

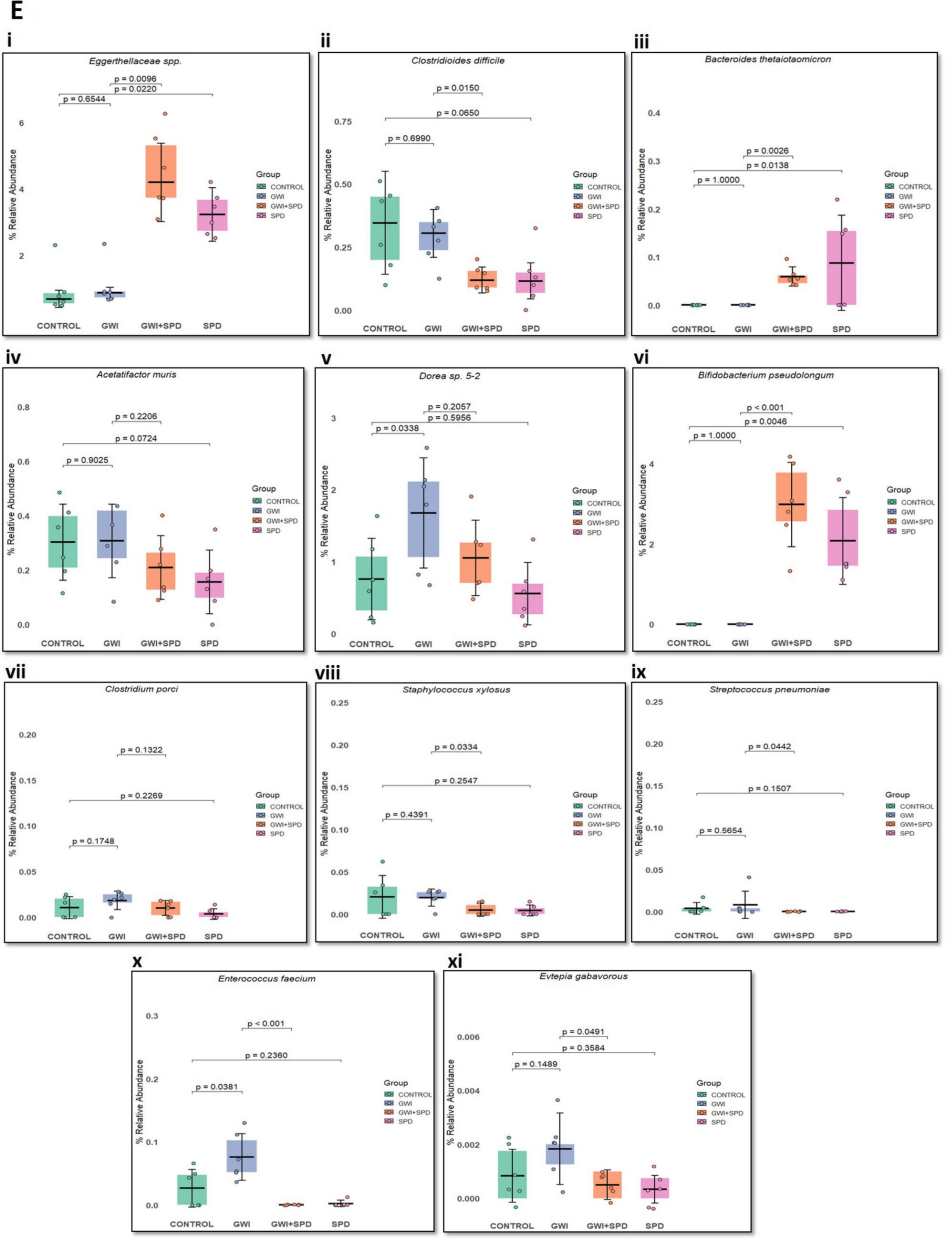
**Spermidine restores gut microbial diversity and composition in a gulf war illness mouse model**. **A.** Relative abundance of gut microbial phyla across experimental groups. Stacked bar plots represent the % relative abundance of taxonomic composition of fecal microbiota at the phylum level across control, GWI, GWI + SPD, and SPD-only groups (*n* = 6 biological replicates/group). **B.** Box plots represent α-diversity (Chao1 index) in fecal samples from each group (*n* = 6 biological replicates/group). Statistical significance was calculated using the Kruskal–Wallis test with pairwise Wilcoxon rank-sum tests as post hoc comparisons (*p* < 0.05). **C.** Box plots represent α-diversity (Shannon index) in fecal samples from each group (*n* = 6 biological replicates/group). Statistical significance was calculated using the Kruskal–Wallis test with pairwise Wilcoxon rank-sum tests as post hoc comparisons (*p* < 0.05). **D.** Principal coordinate analysis (PCoA) plot representing microbial β-diversity (Bray–Curtis dissimilarity) in fecal samples from each group (*n* = 6 biological replicates/group). The plot displays PC1 (49.33% variance) and PC2 (39.36% variance), showing significant distinct clustering of microbial communities. Statistical significance was calculated using permutational multivariate analysis of variance (PERMANOVA), with significance determined at *p* < 0.05 threshold. **E (i–xi).** Differential abundance of key microbial taxa between control, GWI, GWI + SPD, and SPD groups. Box plots illustrate differential % relative abundance of specific bacterial taxa—*Eggerthellaceae* spp., *Clostridioides difficile*, *Bacteroides thetaiotaomicron*, *Acetatifactor muris*, *Dorea *sp. 5-2, *Bifidobacterium pseudolongum*, *Clostridium porci*, *Staphylococcus xylosus*, *Streptococcus pneumoniae*, *Enterococcus faecium*, and *Evtepia gabavorous* between control, GWI, GWI + SPD, and SPD groups (*n* = 6 biological replicates/group). Statistical analysis was determined by Kruskal–Wallis test to compare differences between groups with pairwise Wilcoxon rank-sum tests as post hoc comparisons. Data is represented as mean ± SD and *p* < 0.05 was considered as statistically significant

Whole genome shotgun sequencing (WGS) and taxonomic profiling using mouse fecal samples was performed to study spermidine’s effect on gut microbial diversity and composition in a persistent GWI mouse model (Fig. 1). Figure 1A highlights the % relative abundance of gut microbiota at the phylum level across four groups: control, GWI, GWI + SPD, and SPD. Descriptively, GWI mice exhibit a decreased abundance of *Bacteroidetes* phyla (60.62%) along with an increase in *Firmicutes* (37.51%) compared to the control group (*Bacteroidetes*- 75.95%; *Firmicutes- *22.29%), indicating a shift toward an imbalanced microbial community associated with inflammation and metabolic dysfunction [5]. In contrast, GWI mice treated with spermidine (GWI + SPD) showed a distinct microbial composition with a partial increase of *Bacteroidetes* (63.11%) and a reduction in *Firmicutes* abundance (28.63%), suggesting that spermidine aimed to reestablish phylum-level equilibrium within the gut microbiota. In the SPD-only group, the relative abundances were approximately 25.58% for *Bacteroidetes* and 67.98% for *Firmicutes*. Further, a unique signature abundance of *Actinobacteria* and *Proteobacteria* phyla was observed only in the GWI + SPD and SPD groups. The *Actinobacteria* phylum showed a marked increase in relative abundance in the GWI + SPD (7.50%) and SPD mice (5.34%), with substantially lower levels observed in the control (0.93%) and GWI (1.05%) mice. Similarly, the relative abundance of the *Proteobacteria* phyla was 1.09% in the SPD and 0.76% in the GWI + SPD mice, while it was absent in control and GWI mice. Alpha diversity was represented using both the Chao1 richness estimator and Shannon diversity index (Fig. 1B, C). The Chao1 index estimates species richness by accounting for the number of rare or low-abundance species in the community, while the Shannon index evaluates both richness and species evenness (the relative distribution of species). Spermidine supplementation significantly increased both Chao1 and Shannon indices, highlighting its potential to restore gut microbial richness and evenness (Fig. 1B,* p* = 0.0049 [vs. GWI]; Fig. 1C,* p* = 0.0022 [vs. GWI]). While the differences in alpha diversity between the control and GWI groups were not statistically significant, a visual representation of the data shows slightly lower Chao1 diversity in GWI mice (Fig. 1B,* p* = 0.32 [vs. control]). These findings were consistent with observations in the gut microbiome profile of Gulf War veteran cohorts, which likewise reported no significant changes in alpha diversity but demonstrated a comparable downward trend [3, 5]. This suggests that the overall trend of slightly lower alpha diversity observed in our preclinical mouse GWI cohort matches what others have reported in human GWI microbiome studies. Furthermore, the SPD group also showed significantly higher Chao1 and Shannon diversity with respect to the control groups (Fig. 1B,* p* = 0.0049 [vs. control]; Fig. 1C,* p* = 0.005 [vs. control]). Beta diversity measures differences in microbial community composition between groups. In other words, it is a measure of analyzing similarity and dissimilarity between communities. This was analyzed using PCoA of Bray–Curtis dissimilarity which showed distinct separation of microbial communities across groups, with PC1 (49.33%) and PC2 (39.36%) of the total variance. The GWI + SPD group formed a cluster significantly distinct from the GWI (Fig. 1D, *p* = 0.003), reflecting a unique overall community composition following spermidine treatment. Further, no difference in the community composition was observed between the control and GWI groups (*p* = 0.256). This shift suggests that spermidine not only enhances local species richness but also reconfigures the broader ecological structure of the gut microbiota.

We further analyzed the differential % relative abundance of individual bacterial species to determine specific taxa affected by spermidine treatment. Spermidine treatment in GWI mice significantly increased the relative abundance of *Eggerthellaceae *spp. (Fig. 1E (i), *p* = 0.0096 [vs. GWI]), *Bacteroides thetaiotaomicron* (Fig. 1E (iii), *p* = 0.0026 [vs. GWI]), and *Bifidobacterium pseudolongum* compared to the GWI group (Fig. 1E (vi), *p* < 0.001 [vs. GWI]). *Eggerthellaceae *spp. and *Bacteroides thetaiotaomicron* are associated with beneficial metabolic functions such as bile acid transformation, short-chain fatty acid production, and mucosal barrier support [42, 43] whereas *Bifidobacterium pseudolongum* plays an important role in alleviating diarrhea [44], maintaining gut intestinal homeostasis by suppressing inflammation [45] and regulating secretion of beneficial metabolites like acetate [46]. In contrast, we observed that in GWI, the relative abundance of *Clostridioides difficile* was elevated; however, spermidine administration significantly reduced its relative abundance in GWI + SPD mice (Fig. 1E (ii), *p* = 0.0150 [vs. GWI]). The relative abundance of *Clostridioides difficile* was also significantly decreased in the SPD group compared to the control (*p* = 0.0650). *Clostridioides difficile* is a known opportunistic pathogen responsible for colon infection leading to diarrhea and intestinal mucosal damage [47]. Additionally, spermidine treatment in GWI mice also resulted in decreased relative abundance of *Acetatifactor muris* (Fig. 1E (iv), *p* = 0.2206 [vs. GWI]), *Dorea* sp. 5-2 (Fig. 1E (v), *p* = 0.2057 [vs. GWI]), and *Clostridium porci* (Fig. 1E (vii), *p* = 0.1322 [vs. GWI]) compared to the GWI group; however, these differences were not statistically significant. Interestingly, the % relative abundance of *Dorea *sp. 5-2 was significantly higher in GWI mice compared to that of the control mice (*p* = 0.0338). *Acetatifactor muris* has been reported to hydrolyze conjugated bile acids and use Mucin-2 [MUC2] as a carbon source, thereby contributing to intestinal barrier disruption [48] whereas *Dorea *sp. 5-2 has been associated with fiber fermentation and gas production leading to stomach bloating and discomfort.

Furthermore, we also observed a significant decrease in the % relative abundance of several other bacterial species including *Staphylococcus xylosus*, *Streptococcus pneumoniae*, *Enterococcus faecium*, and *Evtepia gabavorous* in GWI + SPD mice compared to GWI mice (Fig. 1E (viii), *p* = 0.0334 [vs. GWI]; (ix), *p* = 0.0422 [vs. GWI]; (x), *p* < 0.001 [vs. GWI]; (xi), *p* = 0.0491 [vs. GWI]). GWI mice did not show any significant change in the relative abundance of these species with respect to the control except *Enterococcus faecium* (Fig. 1E (x), *p* = 0.0381 [vs. control]). However, SPD mice also showed a significantly lower abundance of these species compared to the control group (Fig. 1E.). *Staphylococcus xylosus* has been well implicated in murine skin infections and is a commensal pathogen [49] whereas *Streptococcus pneumoniae* (pneumococcus) is primarily a colonizer of the upper respiratory tract, but it can also be detected in the gastrointestinal tract, where its interaction with resident microbiota may facilitate opportunistic colonization or infection [50]. *Enterococcus faecium* abundance has been largely associated with an increased inflammatory phenotype in various disease conditions like ulcerative colitis [UC] [51] and IBS [52]. *Evtepia gabavorous* has recently emerged as “γ-aminobutyric acid (GABA)–eating bacteria” [53] because of its potential to efficiently metabolize GABA, an inhibitory neurotransmitter, indicating its role in immune modulation via the gut–brain axis [54]. Moreover, GWI mice exhibited increased abundances of *Turicibacter *spp. and *Extibacter muris*, taxa known for producing secondary bile acids that influence host metabolic and inflammatory responses [55, 56], when compared with control mice (Supplementary Fig. 1A, *p* = 0.6386 [vs. control]; Supplementary Fig. 1B, *p* = 0.1424 [vs. control]). However, spermidine-administered GWI mice showed a significantly decreased abundance of these bacterial taxa compared to GWI (*Turicibacter *spp.: *p* = 0.0058 [vs. GWI]; *Extibacter muris: p* = 0.0109 [vs. GWI]). Finally, a non-significant decrease in *Ruminococcus gnavus* was observed in GWI + SPD mice compared with GWI mice (Supplementary Fig. 1C, *p* = 0.3961 [vs. GWI]). In contrast, this species was significantly increased in GWI mice compared with control mice (*p* = 0.0359). The SPD group also showed a non-significant reduced relative abundance of these three bacterial species with respect to the control (Supplementary Fig. 1). Overall, these findings indicate that spermidine treatment was associated with increased alpha diversity and shifts in global beta diversity, along with the enrichment of beneficial taxa and reduction of potentially pathogenic species, indicating microbiome modulation, thereby suggesting a broader, disease-independent impact of spermidine on the gut microbiome.

### Spermidine Restores Intestinal Barrier Integrity and Inhibits Gut-Derived HMGB1 Release into Systemic Circulation in GWI Mice

**Fig. 2:**
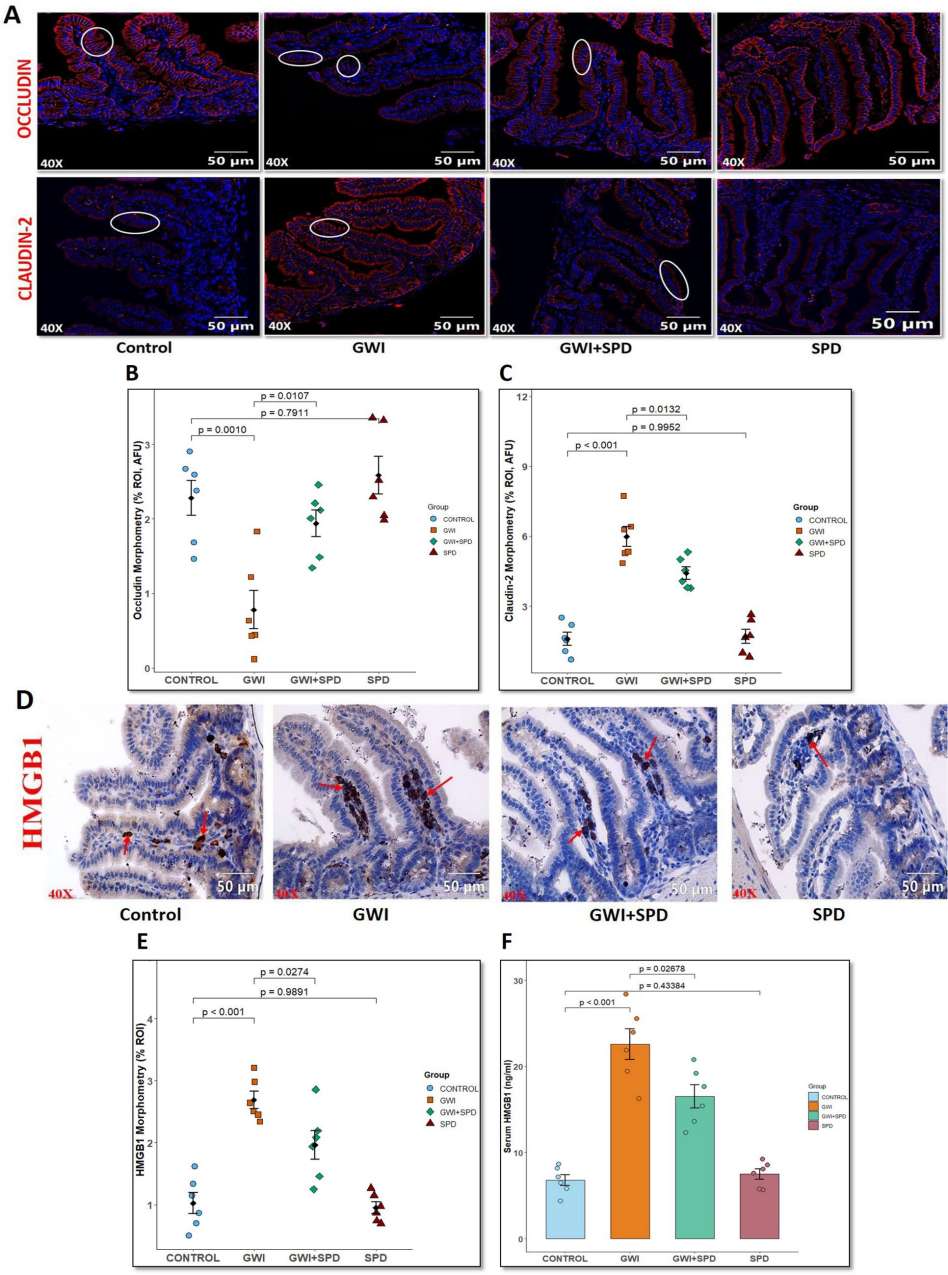
**Spermidine restores intestinal barrier integrity and reduces HMGB1 expression in the small intestine of GWI mice**. **A.** Representative immunofluorescence microscopic images of small intestine tissue sections stained for tight junction proteins occludin (top panel, red) and claudin-2 (bottom panel, red), with DAPI counterstaining (blue) for control, GWI, GWI + SPD, and SPD-only groups. Images were acquired at 40× magnification (scale bar = 50 μm). **B.** Scatter dot plot representing fluorescent intensity of occludin in each group. Quantification was based on immunoreactivity from six distinct microscopic fields per group (*n* = 6 per group). *p*-value from Shapiro–Wilk normality test was 0.348 and for Levene’s test for homogeneity of variance was 0.9032. **C.** Scatter dot plot representing fluorescent intensity of claudin-2 in each group. Quantification was based on immunoreactivity from six distinct microscopic fields per group (*n* = 6 per group). *p*-value from Shapiro–Wilk normality test was 0.2939 and for Levene’s test for homogeneity of variance was 0.5486.** D.** Representative bright-field microscopic images of small intestine sections stained for HMGB1 via immunohistochemistry for control, GWI, GWI + SPD, and SPD-only groups. Images were captured at 40× magnification (scale bar = 50 μm). **E.** Scatter dot plot representing HMGB1 immunoreactivity in each group. Quantification was based on immunoreactivity from six distinct microscopic fields per group (*n* = 6 per group). *p*-value from Shapiro–Wilk normality test was 0.9834 and for Levene’s test for homogeneity of variance was 0.3835. **F.** Bar graph represents serum HMGB1 levels measured using ELISA for control, GWI, GWI + SPD, and SPD-only groups (*n* = 6 biological replicates/group). *p*-value from Shapiro–Wilk normality test was 0.9996 and for Levene’s test for homogeneity of variance was 0.03475. Statistical significance was determined using one-way ANOVA followed by Tukey’s post hoc test or Welch’s ANOVA with Games–Howell post hoc test. *p* < 0.05 is considered statistically significant

Following the observed gut microbial dysbiosis, we next examined whether these microbial alterations resulted in compromised intestinal epithelial barrier integrity and the subsequent release of proinflammatory DAMPs, such as HMGB1. Immunofluorescence staining for essential tight junction (TJ) proteins of epithelial barrier integrity—occludin and claudin-2—was performed (marked by white circles). GWI mice demonstrated severely disrupted barrier structure—decreased occludin and increased claudin-2 immunoreactivity, particularly in the apical regions of the intestinal villi (Fig. 2A, B, *p* = 0.0010 [vs. control]; Fig. 2C, *p* < 0.001 [vs. control]). Remarkably, GWI + SPD mice exhibit restored occludin (*p* = 0.0107 [vs. GWI]) and claudin-2 expression compared to GWI mice (*p* = 0.0132 [vs. GWI]). This shows that spermidine ameliorates intestinal barrier integrity by restoring TJ protein expression in GWI mice. No changes in TJ protein expression were observed in the SPD-only group with respect to the control group (occludin: *p* = 0.7911 [vs. control]; claudin-2: *p* = 0.9952 [vs. control]). We next evaluated the expression of HMGB1, a nuclear protein that acts as a potent DAMP when released in an extracellular milieu.

Immunohistochemical staining of intestinal tissues (Fig. 2D) revealed low baseline HMGB1 immunoreactivity (marked by red arrows) in control mice. In GWI mice, there was a prominent increase in extracellular HMGB1 immunoreactivity (Fig. 2D, E, *p* < 0.001 [vs. control]), indicating active translocation and release of HMGB1 due to epithelial stress or damage. In contrast, GWI + SPD mice showed reduced HMGB1 immunoreactivity (*p* = 0.0274 [vs. GWI]), highlighting that spermidine suppresses HMGB1 secretion from the intestinal epithelium in GWI mice. No significant difference in HMGB1 immunoreactivity was observed between SPD and control group (*p* = 0.9891 [vs. control]). Further, we assessed whether this gut-derived HMGB1 is being released into systemic circulation. We observed that GWI mice exhibited significant increase levels of circulating HMGB1 compared to control mice (Fig. 2F, *p* < 0.001), indicative of increased HMGB1 translocation from intestinal tissue into the circulation. The GWI + SPD group, on the other hand, showed a significant reduction in serum HMGB1 levels (*p* = 0.02678 [vs. GWI]). The SPD group showed no significant change in the serum HMGB1 levels with respect to control (*p* = 0.43384 [vs. control]), demonstrating that spermidine effectively attenuates HMGB1 release from intestinal tissue into circulation.

### Gut-Derived HMGB1 Disrupts the Blood–Brain Barrier and Promotes Microglial Activation via HMGB1-RAGE Signaling in GWI Mice

**Fig. 3:**
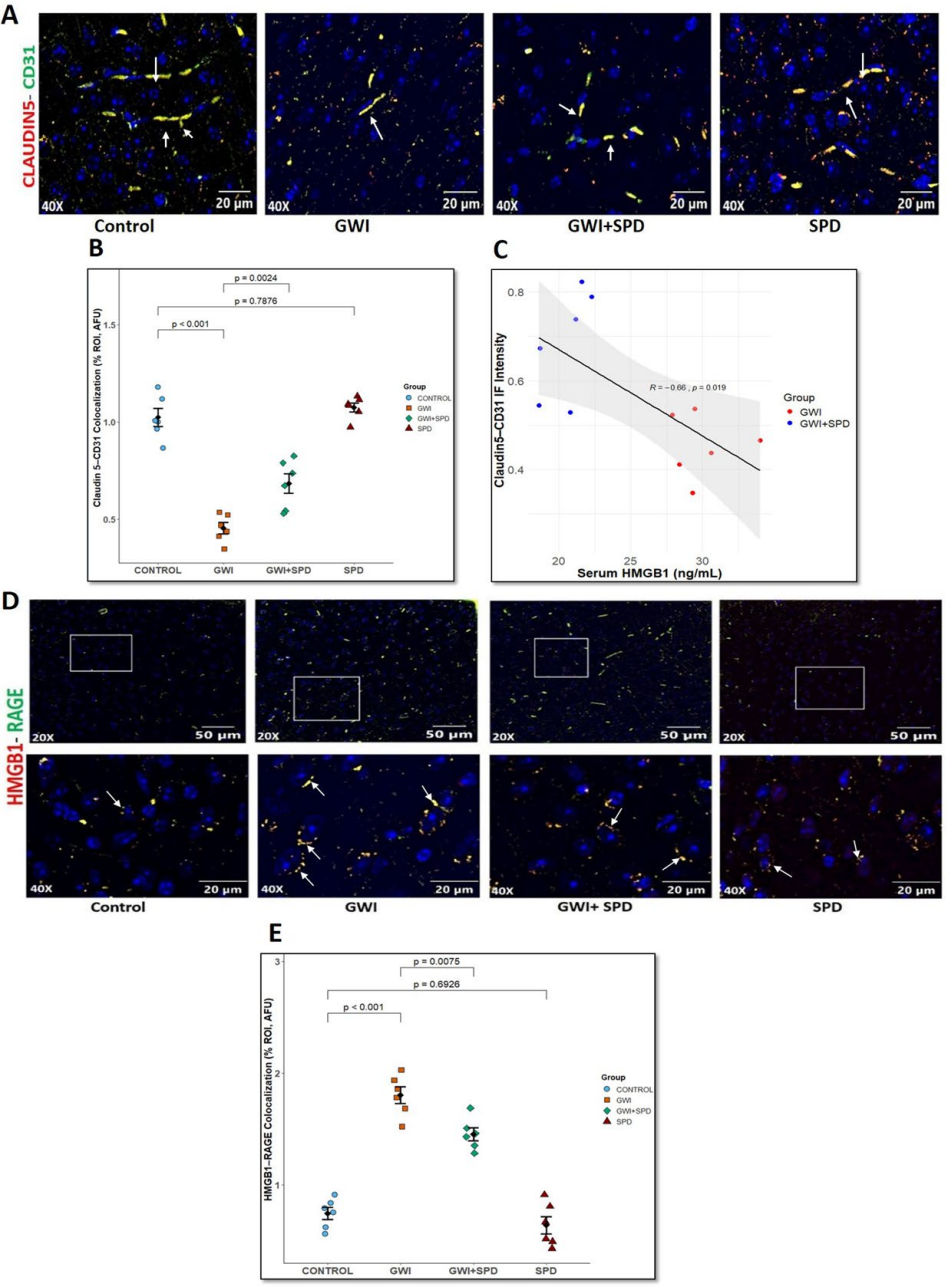

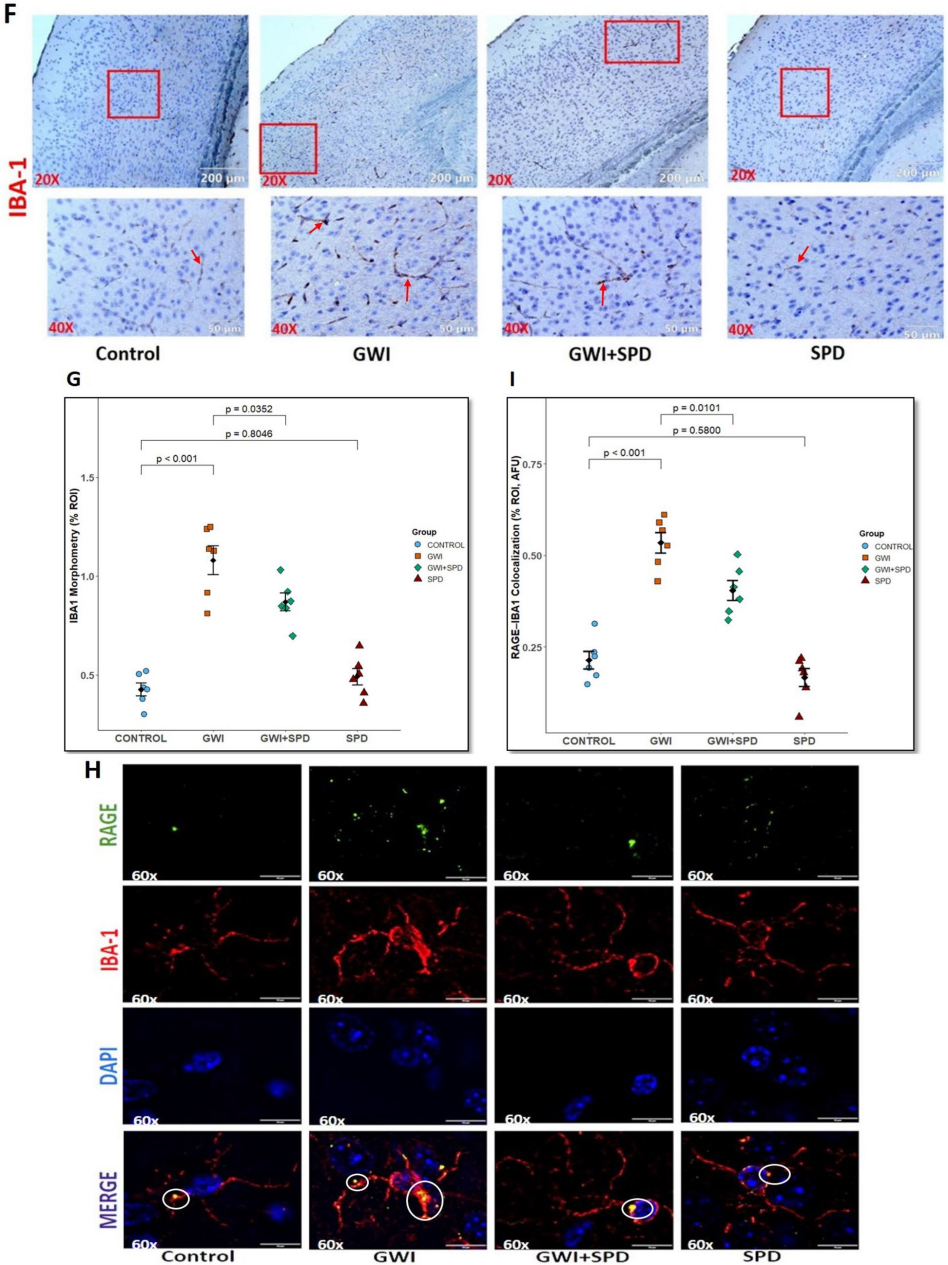
**Spermidine restores blood–brain barrier integrity and reduces HMGB1-RAGE–mediated microglial activation in the prefrontal cortex region of the brain in GWI mice**. **A.** Representative immunofluorescence microscopic images of prefrontal cortex region showing colocalization (represented by yellow color) of claudin-5 (red) and CD31 (green), markers of endothelial tight junctions and vasculature for control, GWI, GWI + SPD, and SPD-only groups. Images were captured at 40× magnification (scale bar = 20 μm). **B.** Scatter dot plot representing colocalization events of claudin-5/CD31 across all groups. Quantification was based on immunoreactivity from six distinct microscopic fields per group (*n* = 6 per group). *p*-value from Shapiro–Wilk normality test was 0.6535 and for Levene’s test for homogeneity of variance was 0.2478. **C.** Linear regression analysis showing a significant inverse correlation between serum HMGB1 levels and claudin-5/CD31 colocalization (*R* = −0.6, *p* = 0.019), supporting a link between systemic HMGB1 and BBB disruption. **D.** Representative immunofluorescence microscopic images of the prefrontal cortex region showing colocalization (represented by yellow color) of HMGB1 (red) and its receptor RAGE (green) for control, GWI, GWI + SPD, and SPD-only groups. Images were captured at 40× magnification (scale bar = 20 μm). **E.** Scatter dot plot representing colocalization events of HMGB1-RAGE across all groups. Quantification was based on immunoreactivity from six distinct microscopic fields per group (*n* = 6 per group). *p*-value from Shapiro–Wilk normality test was 0.6468 and for Levene’s test for homogeneity of variance was 0.6091. **F.** Representative bright-field microscopic images of the prefrontal cortex region showing IBA1 immunoreactivity for microglial activation for control, GWI, GWI + SPD, and SPD-only groups. Images were captured at 40× magnification (scale bar = 50 μm).** G.** Scatter dot plot representing IBA1 immunoreactivity across all groups. Quantification was based on immunoreactivity from six distinct microscopic fields per group (*n* = 6 per group). *p*-value from Shapiro–Wilk normality test was 0.417 and for Levene’s test for homogeneity of variance was 0.4942. **H.** Representative immunofluorescence microscopic images showing colocalization (represented by yellow color) of RAGE (green) and IBA1 (red), counterstained with DAPI (blue) in the prefrontal cortex region of the brain sections for control, GWI, GWI + SPD, and SPD-only groups. Images were captured at 60× magnification (scale bar = 10 μm). **I.** Scatter dot plot representing RAGE-IBA1 colocalization events across all groups. Quantification was based on immunoreactivity from six distinct microscopic fields per group (*n* = 6 per group). *p*-value from Shapiro–Wilk normality test was 0.6984 and for Levene’s test for homogeneity of variance was 0.8640. All data in graphs are presented as mean ± SEM. Statistical analysis was performed using one-way ANOVA with Tukey’s post hoc test; *p* < 0.05 considered significant

Next, we hypothesized that altered circulating HMGB1 might influence the extent of BBB dysfunction and neuroinflammation via microglial activation. Immunofluorescence staining for CD31 (endothelial marker) and claudin-5 (tight junction protein) in brain vasculature revealed that GWI mice show compromised BBB integrity (marked by white arrows) evident by a significant reduction in claudin-5/CD31 colocalization in the prefrontal cortex region compared to the control group (Fig. 3A, B, *p* < 0.001), whereas spermidine treatment significantly rescued this loss by preserving claudin-5/CD31 expression in the GWI + SPD group (*p* = 0.0024 [vs. GWI]). Furthermore, linear regression analysis demonstrated a significant inverse correlation between serum HMGB1 levels and claudin-5/CD31 colocalization (Fig. 3C, *R* = −0.66, *p* = 0.019), supporting our hypothesis that elevated gut-derived HMGB1 release contributes to BBB breakdown. To understand the mechanistic role of systemic HMGB1 in promoting neuroinflammation, we examined the colocalization of HMGB1 and its cognate receptor—RAGE (marked by white arrows) in the prefrontal cortex region of the brain. In the GWI group, we observed significantly increased HMGB1-RAGE colocalization compared to control (Fig. 3D, E, *p* < 0.001). This significant colocalization suggests active engagement of HMGB1 with RAGE, likely contributing to downstream inflammatory signaling and microglial activation. By contrast, the GWI + SPD group exhibited substantially reduced HMGB1-RAGE colocalization compared to GWI mice (*p* = 0.0075). SPD mice showed no significant difference in HMGB1-RAGE colocalization compared to control (*p* < 0.6926). Next, we also studied microglial activation status using IBA1 (a marker for activated microglia) marker. In GWI brain, IBA1 immunoreactivity (marked by red arrows) was significantly elevated in the prefrontal cortex region compared to control (Fig. 3F, G, *p* < 0.001), whereas spermidine treatment mitigated microglial activation, as indicated by decreased IBA1 immunoreactivity in GWI + SPD brain (*p* = 0.0352 [vs. GWI]). The SPD-only group did not show any significant changes in BBB integrity (*p* = 0.7876 [vs. control]) or microglial activation (*p* = 0.8046 [vs. control]). We also evaluated the mechanism linking HMGB1 to microglial activation by performing dual labeling of RAGE with IBA1 (marked by white circles). GWI mice exhibited robust RAGE-IBA1 colocalization (Fig. 3H, I, *p* < 0.001 [vs. control]), indicating enhanced RAGE-mediated microglial activation while GWI mice treated with spermidine (GWI + SPD) showed noticeably reduced RAGE expression and significantly decreased colocalization with IBA1 compared to GWI mice (*p* = 0.0101). The SPD-alone group also displayed low levels of colocalization, comparable to control (*p* = 0.5800), consistent with minimal baseline microglial activation. Quantitative analysis of microglia morphology revealed significantly lower average branch length in the GWI mice compared to the control (Supplementary Fig. 2A, *p* = 0.0014). Treatment with spermidine (GWI + SPD) resulted in significant restoration of average branch length compared to the GWI (*p* = 0.0399). Also, the maximum branch length was significantly decreased in GWI mice compared to control (Supplementary Fig. 2B, *p* = 0.0024) and spermidine supplementation in GWI mice, although not significant, showed a trend toward increased branching compared to GWI mice (*p* = 0.1035). The SPD-only group exhibited microglial morphology consistent with the control group (Supplementary Fig. 2A, *p* = 0.9287 [vs. control]; Supplementary Fig. 2B, *p* = 0.9997 [vs. control]). These results demonstrate that spermidine attenuates GWI-induced intestinal HMGB1 release and its subsequent translocation into circulation, thereby protecting BBB integrity and suppressing RAGE-mediated microglial activation.

### Spermidine Induces AhR/Nrf2/HO-1 Signaling in the Gut to Limit HMGB1 Release and Attenuate Mechanisms Linked to Neuroinflammatory Activation

**Fig. 4:**
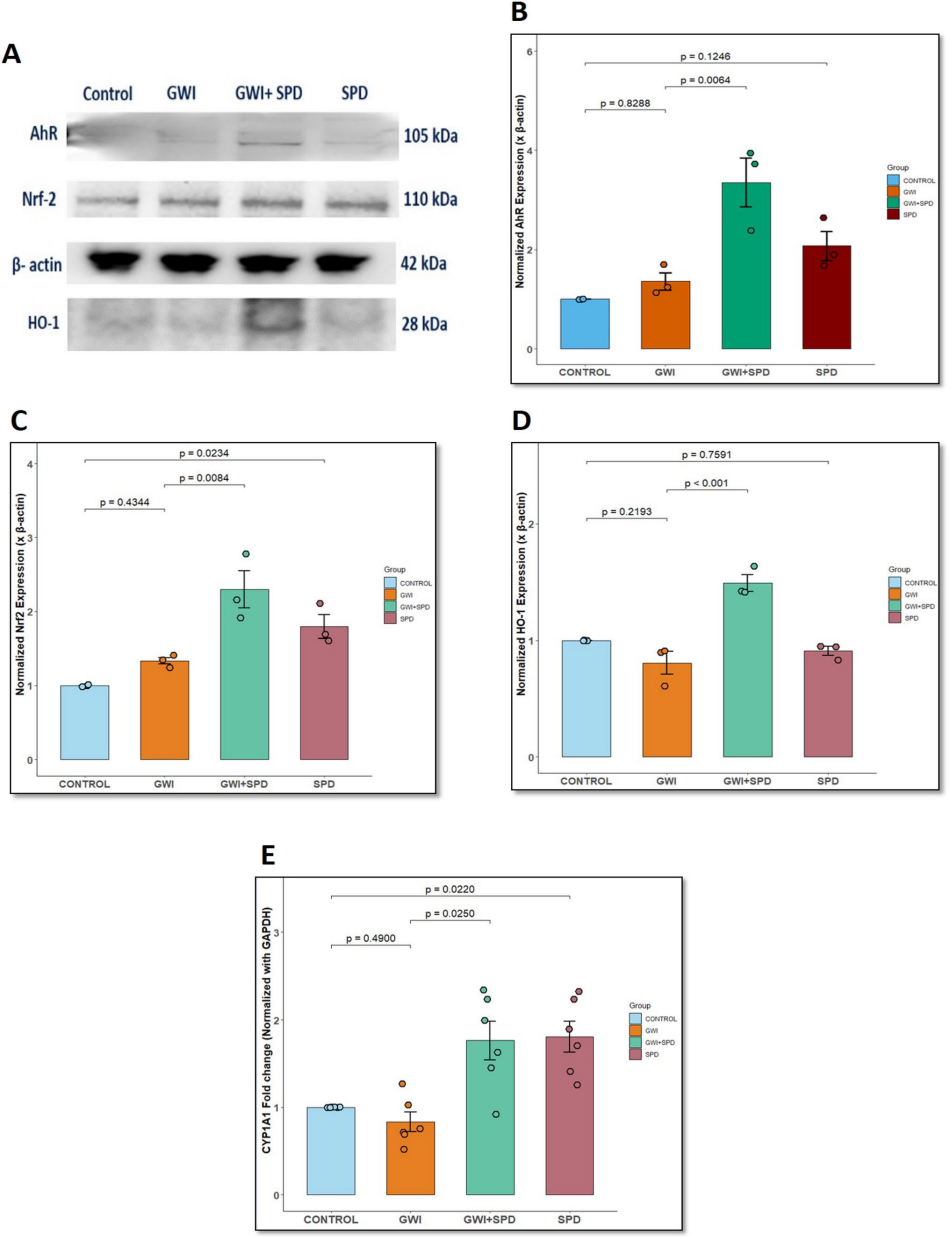
**Spermidine activates AhR/Nrf2/HO-1 signaling in the small intestine of GWI mice to exert its protective effects**. **A.** Representative western blot images showing total protein expression levels of AhR, Nrf2, and HO-1, normalized with β-actin in intestinal tissue lysates from control, GWI, GWI + SPD, and SPD groups. **B.** Bar graph represents densitometric quantification of western blot bands for AhR normalized to β-actin. *p*-value from Shapiro–Wilk normality test was 0.4482 and for Levene’s test for homogeneity of variance was 0.5284. **C.** Bar graph represents densitometric quantification of western blot bands for Nrf2 normalized to β-actin. *p*-value from Shapiro–Wilk normality test was 0.2724 and for Levene’s test for homogeneity of variance was 0.3005. **D.** Bar graph represents densitometric quantification of western blot bands for HO-1 normalized to β-actin. *p*-value from Shapiro–Wilk normality test was 0.8602 and for Levene’s test for homogeneity of variance was 0.6676. **E.** Bar graph represents fold change of CYP1A1 expression analyzed by qRT-PCR in the intestinal tissue of control, GWI, GWI + SPD, and SPD groups (*n* = 6 biological replicates/group). CYP1A1 expression was normalized using GAPDH as endogenous control. *p*-value from Shapiro–Wilk normality test was 0.3325 and for Levene’s test for homogeneity of variance was 0.0060. Data are presented as mean ± SEM. Statistical significance was determined using one-way ANOVA followed by Tukey’s post hoc test or Welch’s ANOVA with Games–Howell post hoc test. *p* < 0.05 is considered statistically significant

The molecular mechanism by which spermidine limits HMGB1 release from the intestinal epithelium and ameliorates neuroinflammation involves AhR activation and its downstream anti-inflammatory signaling molecules—Nrf2 and HO-1 in the small intestine. Activation of Nrf-2 triggers activation of several antioxidant genes, particularly HO-1. HO-1 plays a critical role in maintaining intestinal epithelial homeostasis by limiting oxidative stress and inflammation and also functions as a negative regulator of HMGB1 secretion [45]. HO-1 functions as a protective enzyme possessing anti-inflammatory effects [57] and therefore increased activity of HO-1 inhibits the release of HMGB1 from the nucleus, ultimately inhibiting its active secretion [58]. Densitometric analysis of AhR, Nrf2, and HO-1 protein levels in intestinal tissues revealed that AhR expression remained unchanged in GWI mice compared to control (Fig. 4A, B, *p* = 0.8288). However, GWI mice treated with spermidine exhibited a significant increase in AhR protein expression relative to GWI group (*p* = 0.0064). Although SPD-only mice also showed elevated AhR expression, the data was not statistically significant (*p* = 0.1246 [vs. control]). Similarly, Nrf2 (Fig. 4C, *p* = 0.4344 [vs. control]) and HO-1 protein expression (Fig. 4D, *p* = 0.2193 [vs. control]) did not change in GWI mice with respect to control, but GWI + SPD mice showed a significant increase in both Nrf2 (*p* = 0.0084 [vs. GWI]) and HO-1 protein expression (*p* < 0.001 [vs. GWI]) relative to the GWI group.

CYP1A1 is a canonical target gene of AhR activation and also a reliable molecular marker for AhR transcriptional activity. We selected CYP1A1 to confirm AhR activation in our study because it is not only the most sensitive and specific transcriptional target of AhR, but it also plays well-documented roles in intestinal inflammation and metabolic regulation [59–61] and is robustly expressed when induced throughout the mouse gastrointestinal tract [62]. Our results on the intestinal CYP1A1 mRNA expression revealed no significant change in CYP1A1 expression in GWI mice compared to control mice (Fig. 4E, *p* = 0.4900 [vs. control]), indicating that AhR signaling is not activated in the context of GWI. Contrastingly, GWI + SPD mice demonstrated a significant increase in CYP1A1 mRNA expression relative to GWI mice (*p* = 0.0250). Also, the SPD group showed significant elevated CYP1A1 levels compared to control mice (*p* = 0.0220), suggesting the involvement of spermidine in activating AhR transcriptional activity in the intestinal epithelium.

Together, these findings demonstrate that spermidine activates the intestinal AhR/Nrf2/HO-1 signaling axis, which limits HMGB1 release from the intestinal epithelium into circulation. This reduced intestinal HMGB1 release aligns with the extent of HMGB1-RAGE–mediated BBB disruption and microglial activation shown in Fig. 3, thereby providing a mechanistic link through which spermidine may attenuate downstream neuroinflammatory responses in GWI.

### Spermidine Enhances AhR Nuclear Translocation and Downstream Anti-Inflammatory Signaling in Primary Intestinal Epithelial Cells via AhR/Nrf2/HO-1 Axis

**Fig. 5:**
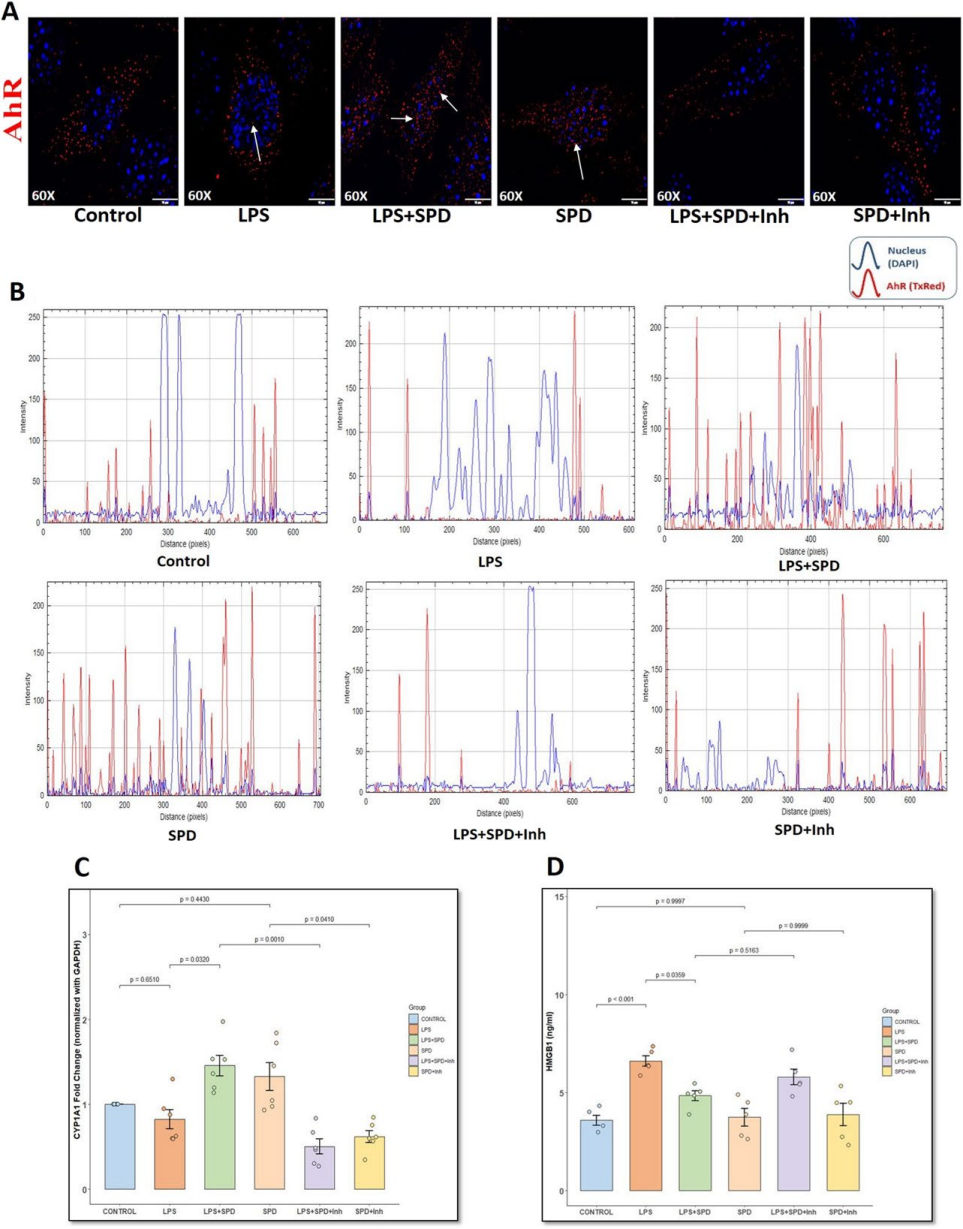

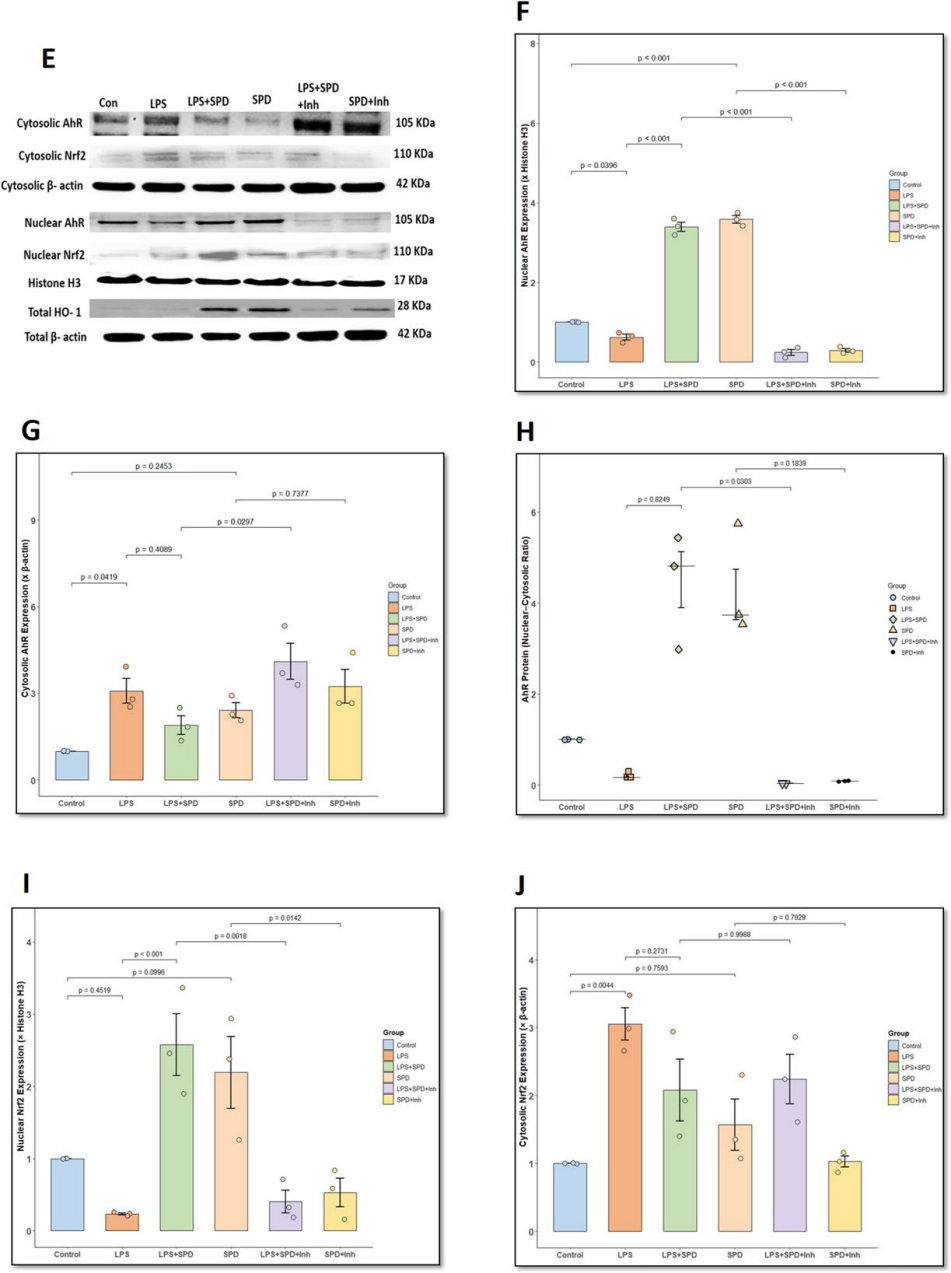

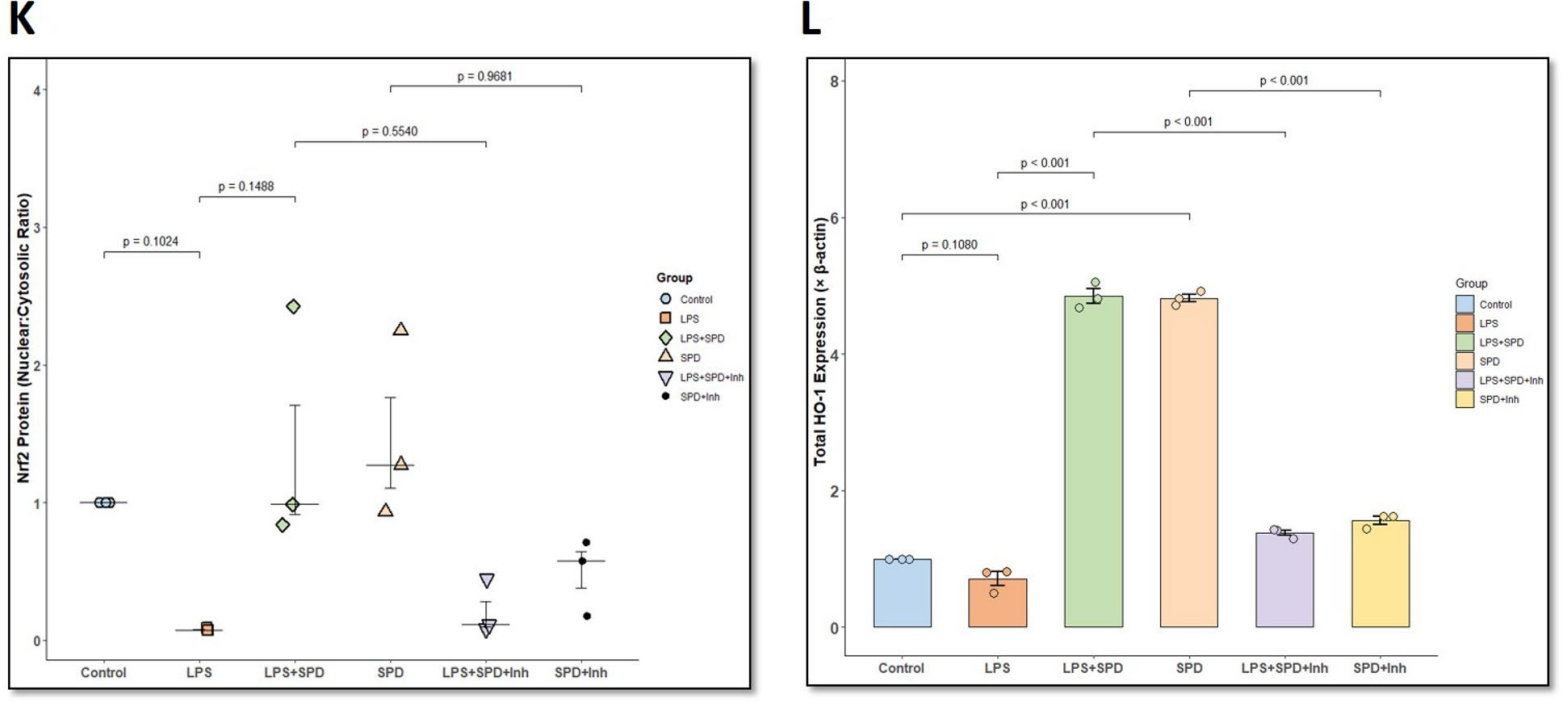
**Spermidine enhances AhR nuclear translocation and downstream transcriptional activation in an AhR-dependent manner in IECs**. **A.** Representative immunofluorescence microscopic images of intestinal epithelial cells (IECs) stained for AhR (red), counterstained with DAPI (blue), showing nuclear translocation of AhR upon spermidine treatment in LPS-stimulated IECs. Images captured at 60× magnification (scale bar = 10 μm). **B.** Line profile intensity plots of DAPI (blue) and AhR (red) fluorescence along defined regions of interest. Line intensity plots showing overlapping peaks of DAPI and AhR confirm the nuclear localization of AhR. Intensity plots were analyzed using FIJI software (ImageJ, version 1.54p). **C.** Bar graph represents fold change of CYP1A1 mRNA expression in IECs treated with spermidine in LPS-stimulated conditions (*n* = 6 replicates/group). CYP1A1 expression was normalized using GAPDH as endogenous control. *p*-value from Shapiro–Wilk normality test was 0.06852 and for Levene’s test for homogeneity of variance was 0.0050. **D.** Bar graph represents HMGB1 levels in the IEC supernatant measured using ELISA for all groups (*n* = 5 replicates/group). *p*-value from Shapiro–Wilk normality test was 0.6969 and for Levene’s test for homogeneity of variance was 0.5918. **E.** Representative western blot images showing cytosolic and nuclear levels of AhR, Nrf2, and total HO-1 protein expression in IECs across treatment groups. Nuclear AhR and Nrf2 protein expression was normalized using Histone H3; cytosolic AhR, Nrf2, and total HO-1 protein expression was normalized using β-actin. **F.** Bar graph represents densitometric quantification of nuclear AhR protein expression normalized to Histone H3. *p*-value from Shapiro–Wilk normality test was 0.6169 and for Levene’s test for homogeneity of variance was 0.4554. **G.** Bar graph represents densitometric quantification of cytosolic AhR protein expression normalized to cytosolic β-actin. *p*-value from Shapiro–Wilk normality test was 0.05633 and for Levene’s test for homogeneity of variance was 0.805. **H.** Scatter dot plot represents nuclear:cytoplasmic ratio of AhR protein expression in IECs across various treatment groups. **I.** Bar graph represents densitometric quantification of nuclear Nrf2 protein expression normalized to Histone H3. *p*-value from Shapiro–Wilk normality test was 0.1851 and for Levene’s test for homogeneity of variance was 0.224. **J.** Bar graph represents densitometric quantification of cytosolic Nrf2 protein expression normalized to cytosolic β-actin. *p*-value from Shapiro–Wilk normality test was 0.2983 and for Levene’s test for homogeneity of variance was 0.4341. **K.** Scatter plot represents nuclear:cytoplasmic ratio of Nrf2 protein expression in IECs across various treatment groups. **L.** Bar graph represents densitometric quantification of western blot bands for total HO-1 protein expression normalized to total β-actin. *p*-value from Shapiro–Wilk normality test was 0.8717 and for Levene’s test for homogeneity of variance was 0.729. Data are presented as mean ± SEM. Statistical significance was determined using one-way ANOVA followed by Tukey’s post hoc test. Statistical difference for nuclear:cytosolic ratio of AhR and Nrf2 protein was determined using Kruskal–Wallis test with Dunn’s post hoc test; *p* < 0.05 is considered significant

Next, we performed in vitro experiments using primary mouse intestinal epithelial cells (IECs) to observe AhR subcellular translocation in response to spermidine treatment. AhR is typically retained in the cytoplasm under basal conditions but translocates to the nucleus upon activation, where it exerts transcriptional control over their downstream targets. As shown in Fig. 5A, immunofluorescence staining, and Fig. 5B, quantitative intensity profile plots, untreated control and LPS-treated IECs showed AhR immunostaining localized largely in the cytoplasmic region, as indicated by the lack of overlap between red and blue peaks in the intensity profile plots (Fig. 5B, control, LPS). Both LPS + SPD and SPD-treated IECs showed pronounced nuclear translocation of AhR (marked by white arrows), evident by overlapping intensity peaks and distinct nuclear colocalization (Fig. 5A, B, LPS + SPD and SPD). Notably, addition of the AhR antagonist, CH223191, markedly reduced nuclear AhR translocation in both LPS + SPD + Inh and SPD + Inh groups (Fig. 5A, B, LPS + SPD + Inh and SPD + Inh), confirming that spermidine facilitates AhR nuclear translocation in intestinal epithelial cells.

We further examined the expression of CYP1A1 gene in IECs under each treatment condition (Fig. 5C). LPS treatment alone did not significantly alter CYP1A1 expression compared to the control (*p* = 0.651). Treatment with SPD alone resulted in a non-significant increase in CYP1A1 mRNA expression compared to control (*p* = 0.4430). However, cotreatment of SPD with LPS upregulated CYP1A1 gene expression compared to LPS-treated cells (*p* = 0.0320 [vs. LPS]), providing mechanistic support for its observed effects in vivo on intestinal AhR signaling. IECs treated with CH223191 showed significantly decreased CYP1A1 expression in both LPS + SPD + Inh (*p* = 0.0010 [vs. LPS + SPD]) and SPD + Inh-treated cells (*p* = 0.0410 [vs. SPD]), further aiding that the observed transcriptional activation was AhR-dependent. In parallel, extracellular HMGB1 levels in IEC supernatants were measured. It was observed that LPS stimulation significantly elevated HMGB1 secretion compared to control (Fig. 5D, *p* < 0.001). Cotreatment with SPD effectively reduced this LPS-induced HMGB1 release (*p* = 0.0359 [vs. LPS]). However, in the presence of AhR inhibitor, HMGB1 levels in both LPS + SPD + Inh and SPD + Inh groups were statistically indifferent compared to LPS + SPD (*p* = 0.5163) and SPD-treated cells (*p* = 0.9999) but remained visually higher than the LPS + SPD and SPD-treated groups. This observation demonstrates that blocking AhR effectively diminishes spermidine’s protective action.

Western blot analysis was performed on treated primary IECs to evaluate spermidine-induced activation of the AhR/Nrf2/HO-1 signaling pathway. To validate the protein expression of AhR and Nrf2 at the subcellular level, nuclear and cytoplasmic fractions were isolated from IECs and probed separately (Fig. 5E). Densitometric quantification highlighted a significant increase in nuclear AhR expression (Fig. 5E, F) in LPS + SPD (*p* < 0.001) compared to the LPS-treated group. On the other hand, the LPS-treated group showed decreased nuclear AhR protein expression compared to the control group (*p* = 0.0396). Cotreatment with the AhR inhibitor CH223191 abrogated this effect, as nuclear AhR expression in LPS + SPD + Inh (*p* < 0.001 [vs. LPS + SPD]) and SPD + Inh groups (*p* < 0.001 [vs. SPD]) were significantly reduced compared to the LPS + SPD and SPD groups. At the cytosolic level (Fig. 5G), LPS treatment showed significantly increased cytosolic AhR expression compared to the control (*p* = 0.0419), and conversely, LPS + SPD cotreatment showed reduced cytosolic AhR expression non-significantly relative to the LPS group (*p* = 0.4089). Cytosolic AhR expression in LPS + SPD + Inh (*p* = 0.0297 [vs. LPS + SPD]) and SPD + Inh groups (*p* = 0.7377 [vs. SPD]) was higher than in the LPS + SPD and SPD groups. To quantify the degree of nuclear translocation, the nuclear:cytosolic (N:C) ratio of AhR was calculated, which showed a non-significant increased ratio in LPS + SPD (Fig. 5H, *p* = 0.8249 [vs. LPS]) compared to LPS-treated cells, reinforcing that spermidine promotes AhR translocation to the nucleus. However, this effect was completely reversed by AhR inhibition in both LPS + SPD + Inh (*p* = 0.0303 [vs. LPS + SPD]) and SPD + Inh groups (*p* = 0.1839 [vs. SPD]), which showed decreased N:C ratio of AhR protein with respect to the LPS + SPD and SPD-treated groups.

Similarly, nuclear Nrf2 expression was significantly increased in the LPS+SPD group compared to the LPS group (Fig. 5E, I, *p* < 0.001). In contrast, nuclear Nrf2 expression in the LPS + SPD + Inh (*p* = 0.0018 [vs. LPS + SPD]) and SPD + Inh groups (*p* = 0.0142 [vs. SPD]) was significantly reduced compared to the LPS + SPD and SPD-treated groups. The nuclear fraction of SPD-treated IECs also showed an increasing trend of Nrf-2 protein expression than control (*p* = 0.0996). Expression of Nrf2 protein in the cytosolic fraction was elevated following LPS treatment (Fig. 5E, J, *p* = 0.0044 [vs. control]); however, a partial non-significant reduction in its expression was observed due to spermidine treatment [LPS + SPD] (*p* = 0.2731 [vs. LPS]). The LPS + SPD + Inh group also showed non-significant elevated cytosolic Nrf2 expression compared to LPS + SPD (*p* = 0.9988 [vs. LPS + SPD]). Also, N:C ratio of Nrf2 protein was elevated in LPS + SPD-treated IECs compared to the LPS-treated group (Fig. 5K, *p* = 0.1488). This activation was decreased in the presence of the AhR inhibitor, CH223191, in LPS + SPD + Inh (*p* = 0.5540 [vs. LPS + SPD]) and SPD + Inh- groups (*p* = 0.9681 [vs. SPD]); however, the data was not statistically significant.

Lastly, HO-1 protein expression was also significantly upregulated in the LPS + SPD group (Fig. 5L, *p* < 0.001 [vs. LPS]), whereas its expression was significantly decreased in the LPS + SPD + Inh group (*p* < 0.001 [vs. LPS + SPD]). Similarly, the SPD-treated group also showed significant increased HO-1 protein expression compared to the control (*p* < 0.001 [vs. control]), whereas its expression was reduced in the SPD + Inh-treated group (*p* < 0.001 [vs. SPD]). No significant change in the expression of HO-1 protein was observed between the LPS-treated IECs and the control (*p* = 0.1080 [vs. control]). Together, these findings confirm that spermidine promotes AhR nuclear translocation and subsequent activation of the Nrf2–HO-1 pathway in intestinal epithelial cells, ultimately leading to a suppression of proinflammatory HMGB1 release.

### HMGB1 Induces RAGE Expression and Microglial Activation in a Dose-Dependent Manner in Immortalized Mouse Microglia [IMG] Cells

Our in vivo findings demonstrated that gut-derived HMGB1 is released into the circulation and is associated with compromised BBB integrity and increased neuroinflammation through microglial activation (Fig. 3). We next sought to determine whether extracellular HMGB1 can directly promote receptor engagement in microglia using the IMG cell line. We intentionally avoided using mouse serum or supernatants from IECs in these experiments. Mouse serum contains a complex mixture of inflammatory mediators, chemokines, hormones, etc. that might confound receptor activation, whereas IEC-conditioned media contained residual lipopolysaccharide (LPS), making it unsuitable for dissecting HMGB1-specific effects. Further, rather than treating cultured IMG cell lines with the HMGB1 concentrations measured in IEC supernatants, we used a broader range of rHMGB1 to evaluate its direct effect on RAGE activation. The commercial rHMGB1 used in this study has a reported ED_50_ ranging from 3 to 25 µg/mL.

**Fig. 6:**
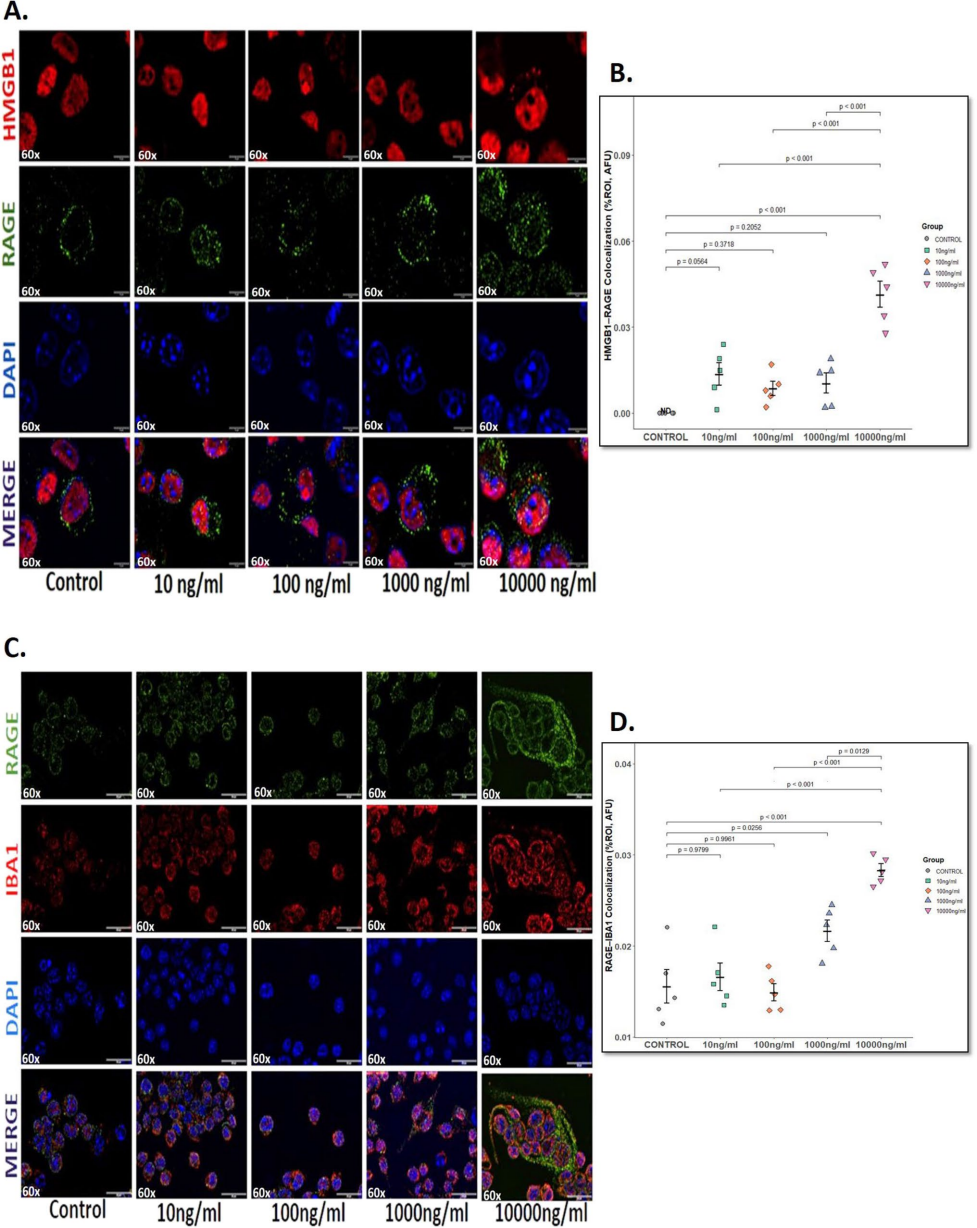
**Dose-dependent HMGB1 induced RAGE expression and microglial activation in IMG cell lines**. **A.** Representative immunofluorescence microscopic images showing colocalization (merged, represented by yellow) of HMGB1 (red) and RAGE (green) in IMG cell lines after treatment with increasing gradient of rHMGB1 (control; 10 ng/mL to 10,000 ng/mL) for 24 h. Nuclei are stained with DAPI (blue). Images acquired at 60× magnification (scale bar = 10 μm). **B.** Scatter dot plot represents HMGB1-RAGE colocalization events in IMG cell lines treated with various concentrations of rHMGB1 (control; 10 ng/mL to 10,000 ng/mL). Quantification was based on immunoreactivity from six distinct microscopic fields per group (*n* = 6 per group). *p*-value from Shapiro–Wilk normality test was 0.4348 and for Levene’s test for homogeneity of variance was 0.1101. **C.** Representative immunofluorescence microscopic images showing colocalization (merged, represented by yellow) of RAGE (green) and IBA1 (red), microglial activation marker, in IMG cell lines following HMGB1 treatment. **D.** Scatter dot plot represents RAGE-IBA1 colocalization events in IMG cell lines treated with various concentrations of rHMGB1 (control; 10 ng/mL to 10,000 ng/mL). Quantification was based on immunoreactivity from six distinct microscopic fields per group (*n* = 6 per group). *p*-value from Shapiro–Wilk normality test was 0.2747 and for Levene’s test for homogeneity of variance was 0.7062. Data are presented as mean ± SEM. Statistical significance was determined using one-way ANOVA followed by Tukey’s post hoc test; *p* < 0.05 is considered significant

IMG cells were treated with rHMGB1 at increasing concentrations (10–10,000 ng/mL) for 24 h. A significant increased HMGB1-RAGE colocalization event was observed only at the highest concentration treatment (10,000 ng/mL) compared to untreated control, 10 ng/mL, 100 ng/mL, and 1000 ng/mL (Fig. 6A, B, *p* < 0.001). The other treatment groups did not show any significant HMGB1-RAGE colocalization with respect to control. This data indicates that higher concentrations of extracellular HMGB1 augment receptor binding, supporting its role as a proinflammatory signaling molecule relevant to neuroinflammation observed in GWI. Next, to determine whether rHMGB1-induced RAGE activation leads to microglial activation, we examined the colocalization of RAGE and IBA1 in the IMG cell line under similar treatment conditions (Fig. 6C, D). Immunofluorescence analysis revealed minimal RAGE-IBA1 colocalization in control and low-dose groups (10–100 ng/mL), whereas cells treated with 1000 ng/mL (*p* = 0.0256 [vs. control]) and 10,000 ng/mL rHMGB1 (*p* < 0.001 [vs. control]) showed robust RAGE expression and evident colocalization with IBA1 compared to control. These findings indicate that extracellular HMGB1 not only promotes RAGE signaling but also leads to downstream activation of microglia, as evidenced by increased RAGE-IBA1 colocalization.

**Fig. 7:**
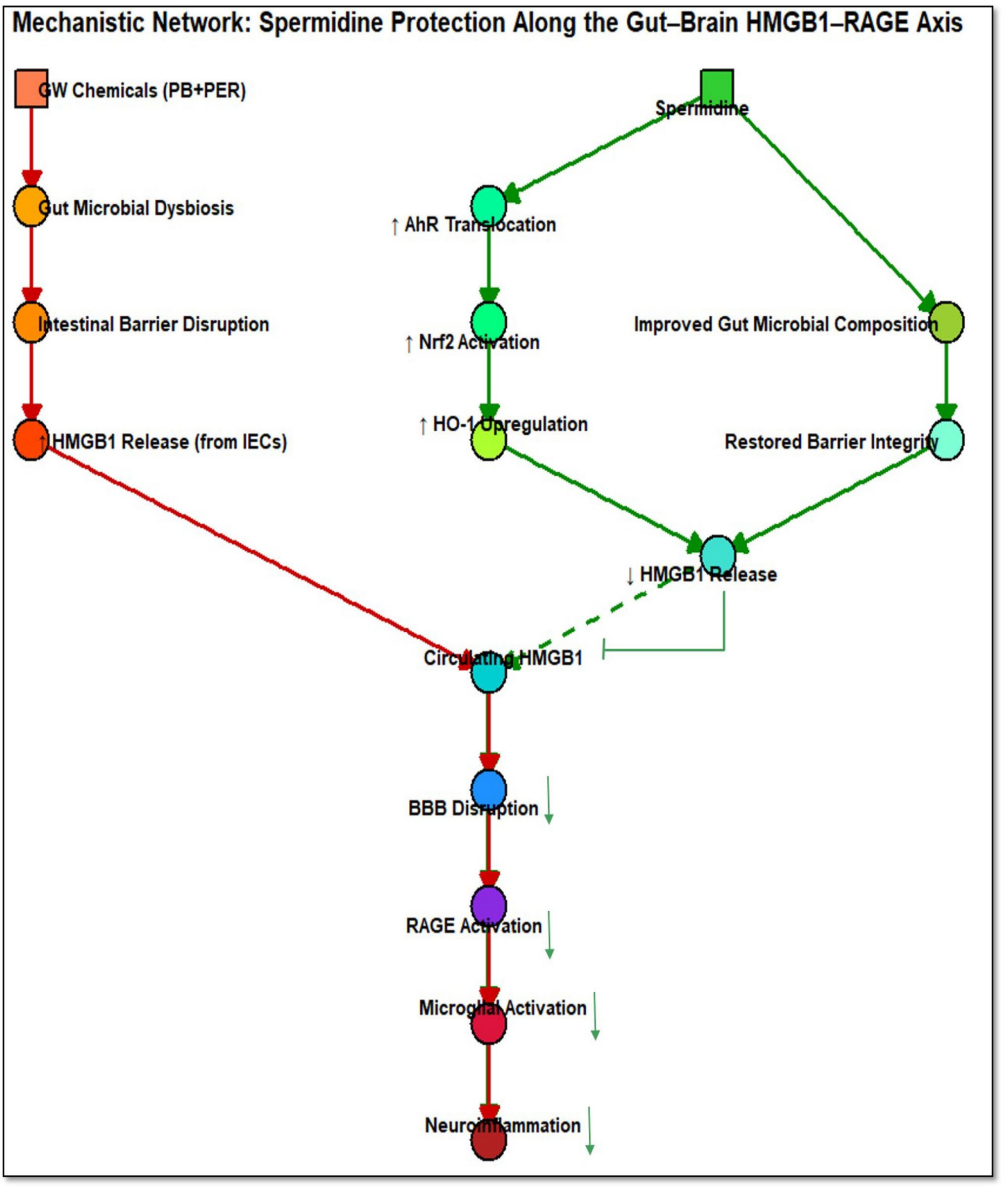
**Mechanistic model illustrating the effects of spermidine on gut–brain axis dysfunction in Gulf War illness (GWI)**. Network represents key molecular and cellular events involved in GWI pathology (yellow/orange color) and spermidine-mediated protective pathways (green color). Exposure to Gulf War chemicals (PB + PER) leads to gut microbial dysbiosis and intestinal epithelial cell (IEC) damage, resulting in intestinal barrier disruption, followed by increased HMGB1 release from IECs into circulation, which disrupts the blood–brain barrier (BBB), activates RAGE in the prefrontal cortex region of the brain endothelium, and promotes microglial activation leading to neuroinflammation. Spermidine treatment improves gut microbial composition and also restores gut barrier integrity, reducing circulating HMGB1 levels by activating AhR/Nrf2/HO-1 signaling pathway in intestinal epithelia. This cascade mitigates downstream neuroinflammatory responses by limiting RAGE activation and microglial reactivity. Red arrows indicate GWI-induced pathogenic events, while green arrows denote spermidine-mediated protective effects. Inhibitory arrow denotes decreased intestinal epithelial HMGB1 release, leading to decreased circulating HMGB1 levels

Lastly, a functional mechanistic network was designed to represent the interplay between GWI pathology and spermidine-mediated effects (Fig. 7). This mechanistic network illustrates the relationship between gut and brain pathology observed in GWI and highlights the therapeutic role of spermidine in mitigating this axis. In the GWI pathway (shown in red arrows), exposure to PB and PER induces an altered microbiome signature that leads to IEC damage and disruption of barrier proteins (occludin and claudin-2). These alterations promote the release of HMGB1 from IECs into circulation, which in turn contributes to BBB disruption, RAGE receptor activation, and microglial activation, ultimately driving sustained neuroinflammation. In contrast, spermidine (green arrows) not only modifies the gut microbiome signature by increasing alpha diversity but counters this pathological cascade by enhancing AhR translocation, Nrf2 activation, and HO-1 upregulation in the intestine. These effects collectively suppress HMGB1 secretion, which reduces its systemic release that prevents downstream BBB disruption and microglial activation, thereby attenuating neuroinflammation. This schematic highlights how spermidine restores gut–brain axis homeostasis by interrupting the HMGB1-mediated inflammatory pathway central to GWI pathology.

## Discussion

Within a year of returning from the 1990–1991 GW, veterans began suffering from a constellation of symptoms, characterized by chronic fatigue, joint and muscle pain, cognitive and memory impairments, skin rashes, gastrointestinal issues, and respiratory difficulties, collectively known as Gulf War Illness. Over the years, growing research has pointed to a likely cause: exposure to harmful chemicals during deployment including pesticides like organophosphates and carbamates, N,N-diethyl-meta-toluamide (DEET), nerve agents such as sarin and cyclosarin, and medications like pyridostigmine bromide, which were given as a protective measure against various chemical attacks [63, 64]. Gut biome alterations and neuroinflammation are characteristic features observed in GWI condition, reported both in clinical and preclinical studies [9, 65–69]. Emerging research has highlighted a bidirectional communication network between gastrointestinal tract and central nervous system (CNS) in linking these two pathologies in GWI [69, 70]. Disturbances in gut microbial composition can influence brain function through neural, immune, and metabolic signaling pathways and inflammatory responses, suggesting that gut dysbiosis may be a key driver of sustained neuro dysfunction in GWI.

Spermidine is a small positively charged aliphatic molecule produced through internal biosynthesis, dietary consumption, and the gut microbiota. It contributes to cellular homeostasis through various physiological processes including cell proliferation, tissue growth and regeneration, regulation of protein translation, and exhibits anti-inflammatory properties [35]. Spermidine also plays a pivotal role in maintaining gut–brain axis integrity by regulating gut microbiota composition and mitigating inflammation, thereby supporting both gut and cognitive health [71]. A previous study from our laboratory reported a significant reduction in spermidine levels in GWI mice, reflected by changes in fecal metabolite profiles [18]. This observation prompted us to investigate the therapeutic potential of spermidine in GWI, particularly with the focus on its role in modulating gut–brain axis dysfunction.

Our data based on whole genome shotgun sequencing demonstrates that spermidine exerts a protective role in GWI by restoring gut microbial diversity and limiting gut-derived inflammation (Fig. 1). We observed that spermidine administration significantly alters both alpha (Chao1 and Shannon) and beta diversity in GWI mice, indicating that spermidine modulated both species richness and abundance distinctly in GWI condition. However, no statistically significant difference in alpha diversity was observed between GWI and control mice; nonetheless, the Chao1 metric showed a visually apparent downward trend in the GWI group. This pattern aligns with findings from studies examining gut microbiome signatures in GW veterans, where 16S rRNA sequencing of stool samples revealed a similar non-significant but consistent reduction in alpha diversity compared to their control counterpart [3, 5], indicating the reliability of our findings. Beta diversity, which is a key metric used to analyze microbial community differences between cohorts, showed statistical distinct clustering of the GWI + SPD group compared to GWI, indicating a significant difference in the overall microbial community structure due to spermidine supplementation. Next, a noteworthy observation was the emergence of *Actinobacteria* and *Proteobacteria* in the spermidine-treated groups (both GWI + SPD and SPD). These phyla were otherwise absent or present in low abundance in both the control and GWI groups. *Actinobacteria* and *Proteobacteria* account for a small percentage of the gut microbiota, but are essential for maintenance of gut homeostasis [72]. *Actinobacteria* are gram-positive bacteria characterized by a high guanine:cytosine [G:C] content in their genomes. Many members of this phylum are known for their extensive capacity to produce secondary metabolites, which contributes to their widespread use as probiotics [73]. Studies on veteran cohorts suffering from GWI have reported an increase in relative abundance of *Actinobacteria* [5, 68]; however, in our study, we did not observe any change in their abundance between GWI and control mice. *Proteobacteria*, on the other hand, are gram-negative bacteria characterized by the presence of LPS in their outer membrane, which contributes to their proinflammatory potential. However, this increase in *Proteobacteria* might be due to the fact that few classes of *Proteobacteria* rely on spermidine for their growth [74]. Also, several other studies have reported that spermidine supplementation increases the relative abundance of *Proteobacteria* species across different disease models such as abdominal aortic aneurysm (AAA) [75] and in animals like silkworm *Bombyx mori* [76]. Further, in our study, we observed decreased abundance of *Bacteroidetes* along with an increase in *Firmicutes* phyla in GWI mice compared to control. Previous studies using persistent preclinical GWI mouse models have not explicitly described the specific trends observed in the *Firmicutes* and *Bacteroidetes* phyla. One study did report an increased abundance of *Firmicutes* relative to *Bacteroidetes*, but this was observed in a subacute GWI mouse model rather than a persistent, long-term model [7]. Similarly, another study examining the relationship between dysbiosis and chronic fatigue in GW veterans also reported an increased abundance of *Firmicutes* and a decreased abundance of *Bacteroidetes* in veterans with GWI compared to deployed veterans without GWI [5]. Collectively, these observations support a shift toward a *Firmicutes*-dominant microbiome in both preclinical and clinical GWI conditions, reinforcing the relevance of our findings.

Next, we also examined the % relative abundance of some relevant short-chain fatty acid (SCFA)–producing and pathogenic bacterial species. We observed increased relative abundance of *Eggerthellaceae *spp., *Bacteroides thetaiotaomicron*, and *Bifidobacterium pseudolongum* and decreased abundance of opportunistic pathogenic bacteria such as *Clostridioides difficile* and *Clostridium porci* in GWI + SPD mice when compared to GWI mice. *Eggerthellaceae *spp. are obligate anaerobic gut-associated bacteria implicated in the regulation of host lipid metabolism and the biotransformation of xenobiotics [77]. Studies have shown that intestinal inflammation observed in UC is associated with lower levels of *Eggerthellaceae *spp., whereas higher levels of these bacteria have been linked to a better disease outcome in such condition [42, 78]. *Bacteroides thetaiotaomicron* is known for its anti-inflammatory and immunomodulatory properties, which help limit pathogen invasion and improve intestinal barrier function via inhibiting the p38 MAPK pathway activation in dextran sodium sulfate (DSS)–induced UC-like colitis preclinical models [79] and acute colitis [80]. *Bacteroides thetaiotaomicron* also plays a key role in nutrient metabolism by directly participating in gut metabolic processes [43]. *Bifidobacterium pseudolongum* plays an important role in lowering triglyceride levels through active lipid metabolism [81] and supports intestinal barrier integrity by activating relevant signaling pathways and modulating gut microbiota composition [45]. *Clostridioides difficile*, on the other hand, has been implicated in severe colon infections; it has the ability to sporulate within the colon and release spores that increase the risk of recurrence and life-threatening complications such as sepsis [47]. Other species whose abundance increased significantly in GWI mice were *Dorea *sp. 5-2 and *Enterococcus faecium* than in control mice. Currently, there is no concrete literature specifically describing the functional role of *Dorea *sp. 5-2, and most available studies characterize *Dorea* at the genus level rather than at the species level [82]. Increased *Dorea *spp. abundance has been consistently reported in patients with IBS and is positively associated with disease severity [83, 84] and similar elevated levels have also been observed in subacute preclinical GWI conditions [7]. *Enterococcus faecium* possesses the inherent ability to develop resistance to various antibiotics and environmental stressors; this trait enables it to thrive as a potent pathogen [85]. This species has been reported to be increased in GW veterans compared to controls [5], demonstrating consistency with our findings. The % relative abundance of both *Dorea *sp. 5-2 and *Enterococcus faecium* was significantly decreased upon SPD administration in treated GWI mice. Moreover, spermidine treatment also significantly attenuated the relative abundance of other taxa that exhibited non-significant upward trends in GWI mice including *Staphylococcus xylosus*, *Streptococcus pneumoniae*, and *Evtepia gabavorous*. *Staphylococcus xylosus* is primarily a murine skin pathogen that shares some of its virulence factors with *Staphylococcus aureus. Staphylococcus xylosus* is capable of forming biofilms, a trait that enhances its ability to persist after initial colonization. Although typically considered a commensal species on murine skin, *Staphylococcus xylosus* can become opportunistically pathogenic when the host immune responses are weakened [49]. *Streptococcus pneumoniae* is a gram-positive bacterium that typically colonizes the upper respiratory tract and is a major causative pathogen of bacterial pneumonia [86]. *Evtepia gabavorous*, which belongs to the Ruminococcaceae family, possesses a unique ability to metabolize GABA, an inhibitory neurotransmitter [54]. It was recently revealed in 2019 that this species lacks the ability to utilize common sugars and amino acids as carbon sources and instead relies on GABA as its primary carbon and energy substrate, suggesting a potential form of gut–brain axis communication through GABA metabolism [87]. To date, no reports have documented the relative abundance change of *Streptococcus pneumoniae* or *Evtepia gabavorous* in a preclinical mouse model of GWI. Our study is the first to identify an increased relative abundance of these taxa in GWI mice, and importantly, spermidine treatment attenuated their levels, suggesting a potential modulatory effect of spermidine on these previously unreported microbial alterations. Lastly, we also observed decreased relative abundance of *Turicibacter *spp., *Extibacter muris*, and *Ruminococcus gnavus* in our spermidine-administered GWI mice compared to GWI mice (Supplementary Fig. 1). These bacterial species were otherwise significantly elevated or demonstrated an upward trend in GWI mice. *Extibacter muris* functions in metabolizing cholic acid (CA), a primary bile acid to deoxycholic acid (DCA), a secondary bile acid via the 7α-dehydroxylation reaction [56] while *Turicibacter *spp. possess bile salt hydrolase (BSH) and 7α-hydroxysteroid dehydrogenase (HSDH) activities that modulate secondary bile acid composition and host lipid metabolism [55]. Therefore, the increased abundance of these bacterial taxa in GWI mice may suggest a potential shift in bile acid metabolism and host lipid regulation, as they possess bile-transforming enzymes that can alter metabolic and inflammatory pathways in the host. Increased abundance of *Ruminococcus gnavus*, on the other hand, is strongly associated with the development of Crohn’s disease and IBD [88]. *Ruminococcus gnavus* is reported to be a proinflammatory bacterial species which synthesizes and secretes inflammatory polysaccharide, glucorhamnan, and also utilizes mucin as its carbon source, contributing to the breakdown of the intestinal epithelial barrier [89]. Overall, these microbiome shifts suggest that spermidine promotes a gut environment favoring beneficial commensals that promote mucosal barrier homeostasis while suppressing pathogenic taxa in GWI conditions. Previous studies on spermidine have reported its potent ability to alleviate gut microbiome signatures not only in colitis models [27] but also in various other diseases such as diet-induced obesity [37] and nonalcoholic steatohepatitis (NASH) [90]. In particular, spermidine has been reported to prevent the dysbiotic shift toward the overgrowth of other pathogenic taxa—*Gammaproteobacteria*, *Escherichia*, *Shigella*, *Enterococcus*, and *Helicobacter* [27]. This data is consistent with our findings, indicating a significant role of spermidine in ameliorating gut dysbiosis; while the specific pathogenic taxa differ, the overall pattern of preventing pathogenic overgrowth and restoring microbial balance aligns with previous studies.

Gut microbiome disturbances leading to compromised intestinal barrier function are well documented in GWI, which promotes release of inflammatory mediators such as HMGB1 into systemic circulation [15]. In our study, spermidine supplementation significantly improved intestinal epithelial integrity, evidenced by increased expression of tight junction protein—occludin—and decreased immunoreactivity of pore-forming protein, claudin-2, and reduced HMGB1 epithelial release (Fig. 2). HMGB1 is a ubiquitous nucleoprotein released into the extracellular space during inflammation. In our study, GWI mice exhibited increased HMGB1 immunoreactivity in the intestinal epithelium and elevated systemic HMGB1 levels, both of which were attenuated by spermidine administration (Fig. 2D–F). These observations suggest that the gut, particularly the intestinal epithelium, may be a significant source of circulating HMGB1 under GWI conditions [15]. Circulating HMGB1 has been identified as a key peripheral factor driving persistent neuroinflammation in GWI, even in the absence of traditional proinflammatory cytokines, both in preclinical and clinical GWI models [13, 16]. HMGB1 also plays a crucial role in disrupting BBB integrity in various neuro-diseased models such as Alzheimer’s disease [91] and acute ischemic stroke [92] as well as GWI [93]. The study by Garza-Lombó et al. [13] demonstrated that HMGB1 alone is sufficient to activate microglia and induce a unique inflammatory gene signature, implicating it as a mechanistic link between peripheral immune dysfunction and long-lasting CNS pathology in GWI clinical and preclinical models. Our findings also revealed sustained microglial activation with increased IBA1 immunoreactivity in the prefrontal cortex of GWI mice and enhanced HMGB1-RAGE interaction. This finding was accompanied by compromised BBB integrity, as observed by reduced claudin-5/CD31 colocalization, that strongly correlated with elevated serum HMGB1 levels. However, spermidine treatment not only restored BBB integrity significantly but also reduced HMGB1-RAGE colocalization, along with attenuated microglial activation in the prefrontal cortex region of the brain (Fig. 3). These results suggest that gut-derived circulating HMGB1 plays a critical role in accelerating BBB disruption and neuroinflammation in GWI and that limiting its release through spermidine administration may help prevent systemic to brain inflammatory signaling and microglial dysfunction.

AhR activation by spermidine induces Nrf2 transcription which promotes its nuclear translocation by dissociation from Keap1, a cytoplasmic inhibitor that targets Nrf2 for degradation [94]. Nrf2 regulates the antioxidant/anti-inflammatory response by inducing heme oxygenase-1 (HO-1) expression. HO-1 functions in the degradation of heme into biliverdin, iron, and carbon monoxide but plays a key role in suppressing inflammation and oxidative damage. HO-1 is also reported to prevent HMGB1 release by blocking its translocation from the nucleus to the cytoplasm [58], thereby exerting anti-inflammatory effect. Spermidine-mediated activation of the AhR/Nrf2 pathway, along with inhibition of STAT3 phosphorylation, has been reported to play a central role in maintaining redox balance and intestinal barrier homeostasis in murine colitis models [25]. AhR activation has also been shown to upregulate ODC1 enzymatic activity, thereby enhancing polyamine biosynthesis, which in turn suppresses NLRP3 inflammasome activation, caspase-1 activation, and gasdermin-D pore formation, ultimately reducing pyroptosis and mucosal inflammation [95]. Furthermore, spermidine also influences kynurenine pathway, where kynurenine, an endogenous AhR ligand, serves as an important intermediary linking tryptophan metabolism to AhR-mediated anti-inflammatory signaling [96]. In addition, spermidine has been reported to regulate the activity of protein tyrosine phosphatase nonreceptor type 2 (PTPN2), a key modulator of immune cell function and intestinal epithelial barrier integrity [27]. Based on this well-documented ability of spermidine in mitigating intestinal pathology, we focused our study on evaluating whether spermidine-mediated AhR activation could mitigate GWI pathology. Mechanistically, our data indicate that spermidine treatment in GWI mice activates the AhR/Nrf2/HO-1 signaling axis in the intestine by significantly upregulating the protein expression of AhR, Nrf2, and HO-1 when compared to GWI mice. This activation ultimately led to reduced HMGB1 release from the intestinal epithelium and decreased microglial activation via HMGB1-RAGE interaction (Fig. 4). Although spermidine robustly increased AhR activation in our model, the exact metabolic mechanism remains uncertain. Spermidine is not a classical AhR ligand; instead, it likely enhances AhR activity indirectly through modulation of tryptophan-like kynurenine metabolism or polyamine-driven anti-inflammatory pathways reported in prior research [97] and the current study did not directly quantify kynurenine metabolism or other candidate ligand pools. Future studies assessing indoleamine 2,3-dioxygenase 1/tryptophan 2,3-dioxygenase 2 (IDO1/TDO2) activity, kynurenine levels, and other endogenous AhR ligands will be necessary to map the precise metabolic route involved in GWI condition. Despite this limitation, our data showing increased CYP1A1 expression provide functional evidence of AhR pathway activation following spermidine administration. CYP1A1 is a direct downstream target of AhR pathway activation at the transcript level, and we observed a significant increase in its expression following spermidine treatment, consistent with AhR signaling activation. Further, to functionally validate spermidine’s role in mitigating GWI pathology via AhR signaling, we employed pharmacological inhibition of AhR using CH223191 in vitro. We used LPS to stimulate primary intestinal epithelial cell lines in our in vitro work. Multiple studies have emphasized that GWI is characterized by innate immune priming, where prior exposure to PB/PER and deployment-related stressors sensitizes immune cells to subsequent inflammatory challenges. In a review article by Trageser et al. [41], the authors discuss how LPS is used experimentally to model innate immune activation and inflammatory priming, a mechanism suggested to mirror how exposure to GW toxins might “prime” the innate immune system [41]. Thus, LPS is not invoked as an actual exposure in GWI but rather as a model system showing how priming + inflammatory challenge can lead to persistent pathology. Another study by Kimono et al. [40] showed that GWI-relevant chemical exposures disrupt the gut microbiome, increase endotoxin (LPS) levels, and impair intestinal epithelial barrier integrity in both subacute and persistent GWI mouse model [7, 40]. Kimono et al.’s in vitro work further demonstrates that LPS-primed epithelial cells show amplified cytokine responses when exposed to enteric glial cells, a pathology similar to GWI pathology in vivo. Spermidine treatment induced a noticeable translocation of AhR from the cytoplasm to the nucleus, indicating its role in AhR signaling activation, and was accompanied by increased protein expression of AhR, Nrf2, and HO-1 in primary IECs. In addition, CYP1A1 levels were also significantly upregulated in primary IECs treated with spermidine, confirming that spermidine’s protective effects are AhR dependent (Fig. 5).

Furthermore, validation in IMG cell lines was assessed using rHMGB1 across a range of concentrations. We evaluated whether extracellular HMGB1 directly engages microglial receptors and observed a significant HMGB1-RAGE colocalization at the highest dose (10,000 ng/mL), indicating HMGB1’s role in RAGE-mediated inflammatory signaling. Lastly, this receptor engagement was confirmed to induce microglial activation, as evidenced by increased RAGE colocalization with IBA1 in a dose-dependent manner (Fig. 6). Collectively, these findings demonstrate that extracellular HMGB1 is a potent driver of RAGE-dependent microglial activation in GWI pathology.

While our study provides compelling mechanistic insights into spermidine’s therapeutic effects in the context of GWI, several limitations should be acknowledged. First, although pharmacological inhibition with CH223191 was used to implicate AhR signaling, the absence of intestinal epithelial cell–specific knockout models (e.g., IEC-specific AhR-deficient mice) limits our ability to definitively attribute the observed gut barrier protection to epithelial AhR. Future studies employing these genetic models will allow us to precisely dissect the cell type–specific contribution of epithelial AhR to gut barrier regulation under GWI conditions. Similarly, the use of IEC-specific HMGB1-deficient mice in future work would help confirm whether the intestine is the primary source of elevated systemic HMGB1 observed in GWI preclinical and clinical models. These mice will allow us to directly test cause-and-effect relationships between gut-derived signals and systemic neuroimmune dysfunction in GWI. Second, as noted earlier, we did not stimulate IMG microglial cells with IEC supernatants because IECs were pre-exposed to LPS. Using these supernatants would have introduced confounding activation of multiple inflammatory pathways, making it challenging to attribute downstream effects specifically to spermidine or AhR modulation. Future studies utilizing LPS-free epithelial stimulation systems (e.g., transwell cocultures or microfluidic epithelial-immune platforms) tomore precisely evaluate epithelial–microglial crosstalk must be performed. Finally, although we provide molecular, immunohistochemical, and cellular evidence of neuroinflammation, the lack of behavioral assessments such as the three-chamber sociability test, open field test, and novel-object recognition test prevents direct correlation of these biological changes with cognitive or social impairments typically observed in GWI. As a defined next step, future studies incorporating standardized behavioral paradigms focused on memory, anxiety, and social interaction should be conducted to directly link gut- neuroimmune alterations with functional behavioral outcomes.

In summary, our findings reveal that spermidine ameliorates GWI-related gut dysbiosis and neuroinflammation via AhR/Nrf2/HO-1 signaling and suppression of gut-derived HMGB1 (Fig. 7). These results support further investigation of spermidine as a promising therapeutic strategy to restore gut–brain axis homeostasis in GWI and related neurological disorders.

## Conclusion

This study investigated the role of spermidine in ameliorating GWI pathophysiology by targeting the gut–brain axis. Using a 22-week persistent GWI mouse model exposed to PB and PER, we demonstrated that spermidine supplementation significantly restored gut microbial diversity and composition via modulating alpha and beta diversity. It also improved phylum-level imbalances and selectively enriched anti-inflammatory and metabolically beneficial taxa such as *Eggerthellaceae* spp., *Bacteroides thetaiotaomicron*, and *Bifidobacterium pseudolongum* while suppressing opportunistic pathogens such as *Clostridioides difficile* and *Enterococcus faecium*. This microbial restoration was closely associated with improved intestinal barrier integrity, reflected by increased TJ protein expression and reduced levels of pore-forming protein. Furthermore, spermidine also suppressed the epithelial release of the proinflammatory alarmin—HMGB1 into the circulation, thereby mitigating systemic inflammatory signaling. In the brain, spermidine preserved BBB integrity and significantly reduced HMGB1-RAGE colocalization in the prefrontal cortex region of the brain, which led to a reduction in microglial activation, demonstrated by decreased IBA1 expression and restored microglial branching morphology. Mechanistically, we observed that spermidine activated intestinal AhR/Nrf2/HO-1 signaling axis, a pathway known for its anti-inflammatory and cytoprotective roles. This activation limited HMGB1 release at the gut level and curtailed downstream neuroimmune activation. These findings were validated in vitro using primary intestinal epithelial cells, where spermidine facilitated AhR nuclear translocation, upregulated CYP1A1 and HO-1 expression, and reduced LPS-induced HMGB1 secretion in an AhR-dependent manner. To further examine the direct role of HMGB1 in brain inflammation, we treated mouse IMG cell lines with rHMGB1. rHMGB1 treatment led to an increase in HMGB1-RAGE colocalization events and also promoted RAGE-IBA1 colocalization at higher concentrations, confirming the potential of HMGB1 in activating microglia through RAGE signaling, which parallels our in vivo findings in GWI mice.

## Supplementary Information

Below is the link to the electronic supplementary material.

ESM 1 (PDF 1.74 MB)

## Data Availability

Entire microbiome sequencing data is available via the Sequence Read Archive (SRA) database, accession ID: **PRJNA1307016** : Spermidine Attenuates Neuroimmune Dysfunction in Gulf War Illness via Modulation of the Gut Brain Axis.
