# Supplementary material for: Spermidine Attenuates Neuroimmune Dysfunction in Gulf War Illness via Modulation of the Gut- Brain Axis
Source: Mol Neurobiol. 2026 Apr 10;63(1):556. doi: 10.1007/s12035-026-05763-6 (PMC13068726; doi:10.1007/s12035-026-05763-6)

FIGURE. 1: SUPPLEMENTARY FIGURE

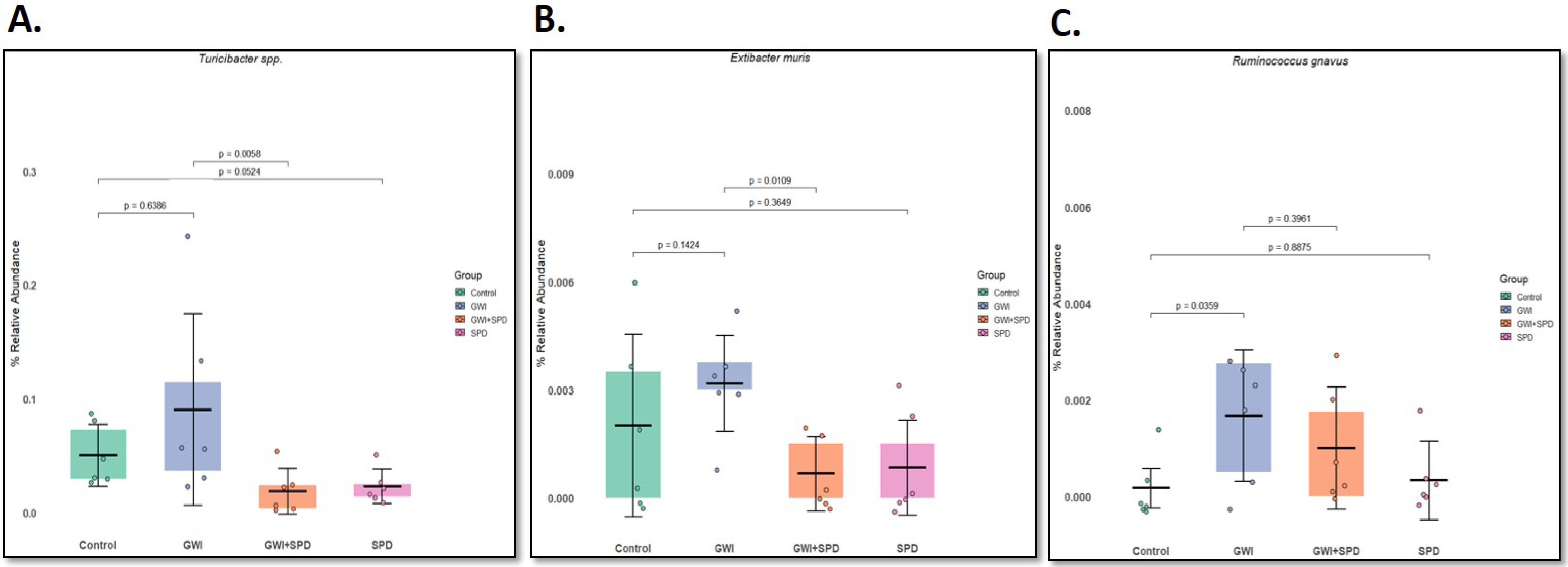

Supplementary Figure 1. Differential abundance of key microbial taxa between Control, GWI, GWI+SPD and SPD groups.

Box plots illustrate differential % relative abundance of specific bacterial taxa- **A.** *Turicibacter spp.,* **B.** *Extibacter muris* and **C.** *Ruminococcus gnavus* between Control, GWI, GWI+SPD and SPD groups (n= 6 biological replicates/ group). Statistical analysis was determined by Kruskal- Wallis test to compare differences between groups. $p < 0.05$ was considered as statistically significant.

FIGURE. 2: SUPPLEMENTARY FIGURE

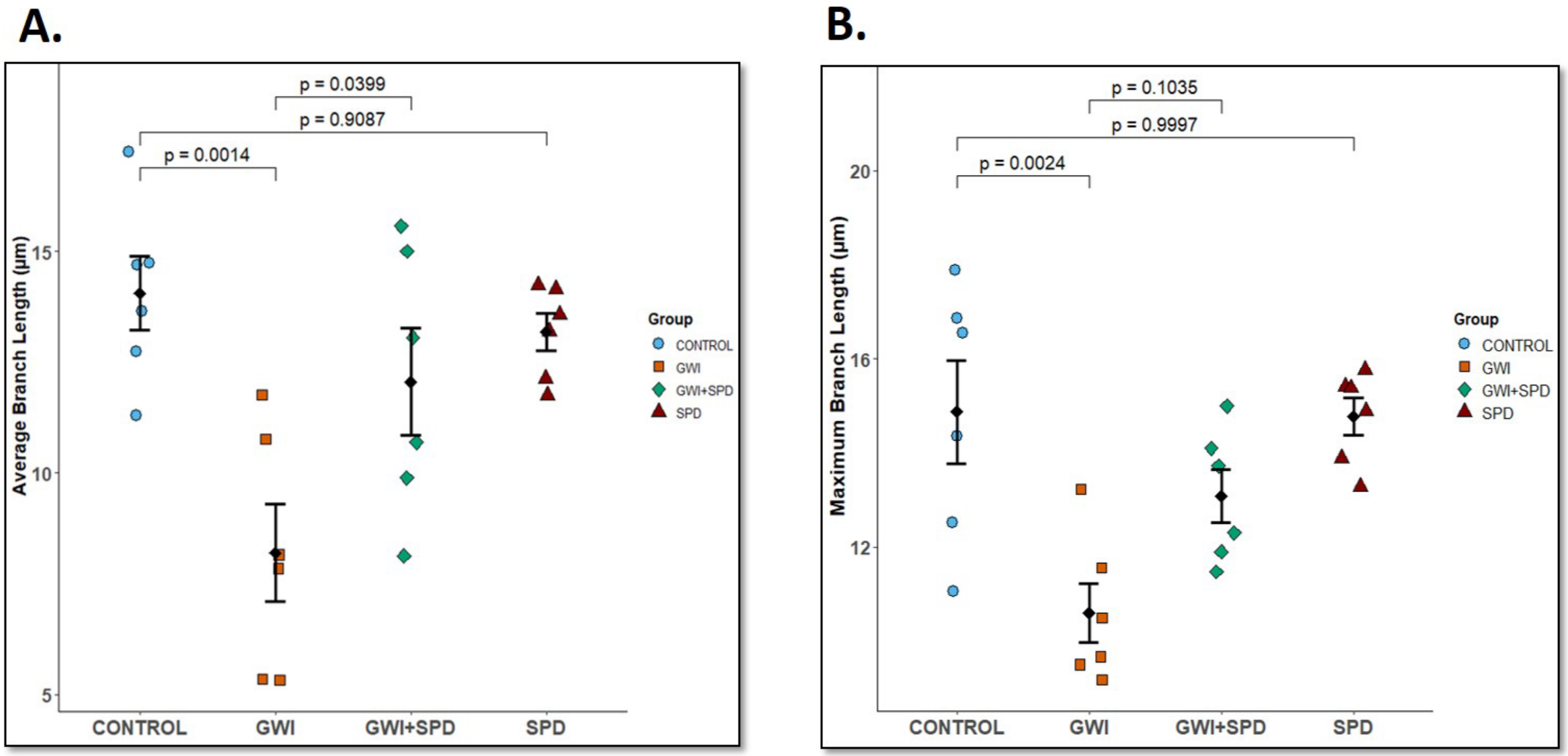

Supplementary Figure 2: Quantitative analysis of microglial morphology in the prefrontal cortex region of the brain.

A. Average branch length of IBA1 microglia across Control, GWI, GWI+SPD and SPD- only groups. Average branch length was calculated using FIJI software (ImageJ, version 1.54p). Quantification was based on 6 distinct microscopic fields per group (n= 6 per group). *p*-value from Shapiro-Wilk normality test was 0.4684 and for Levene's test for homogeneity of variance was 0.1116. **B.** Maximum branch length of IBA1 microglia across Control, GWI, GWI+SPD and SPD- only groups. Maximum branch length was calculated using FIJI software (ImageJ, version 1.54p). Quantification was based on 6 distinct microscopic fields per group (n= 6 per group). *p*-value from Shapiro-Wilk normality test was 0.9016 and for Levene's test for homogeneity of variance was 0.0698. Data are presented as mean ± SEM. Statistical significance was determined by one-way ANOVA with Tukey's post hoc test; $p < 0.05$ considered significant.

WHOLE WESTERN BLOTS

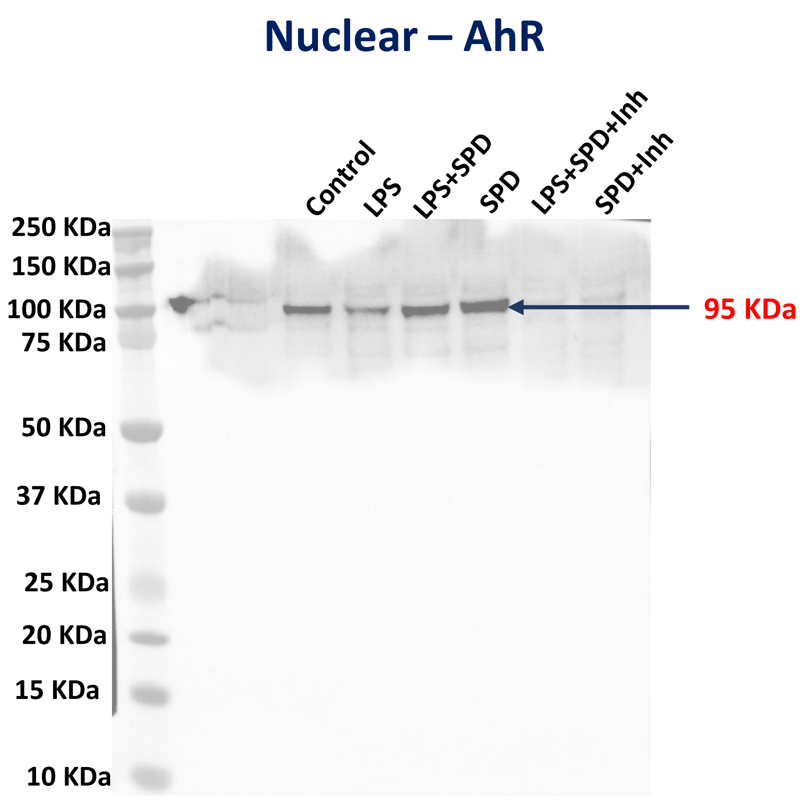

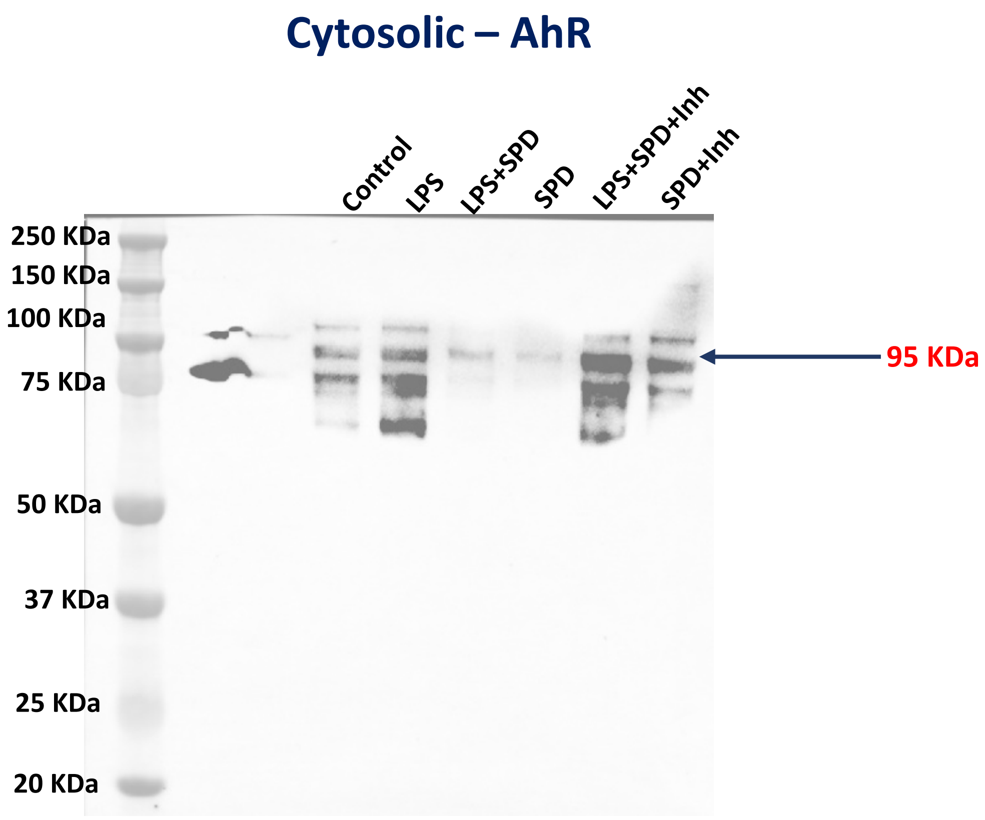

WHOLE WESTERN BLOTS

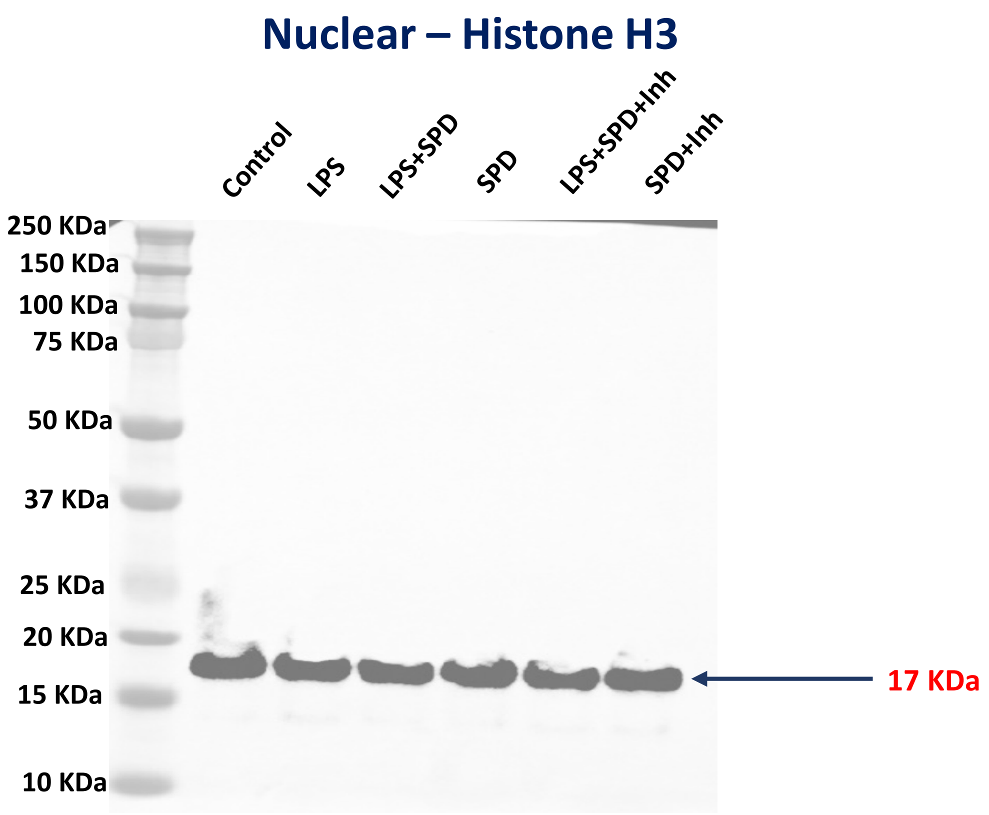

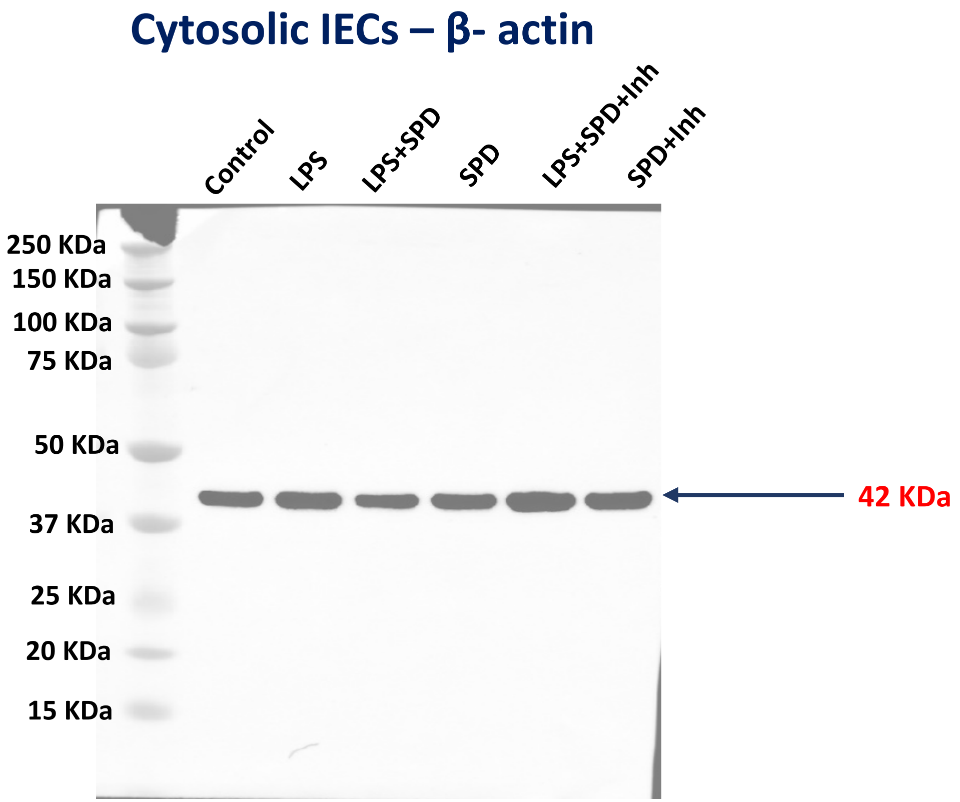

WHOLE WESTERN BLOTS

Nuclear IECs – Nrf2

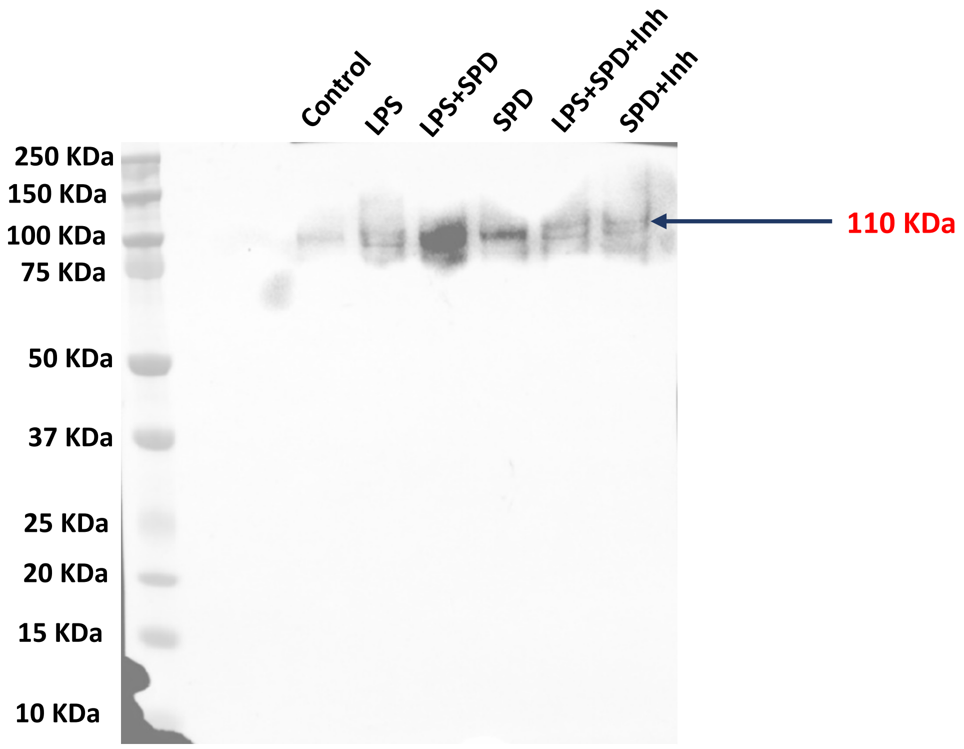

Cytosolic IECs – Nrf2

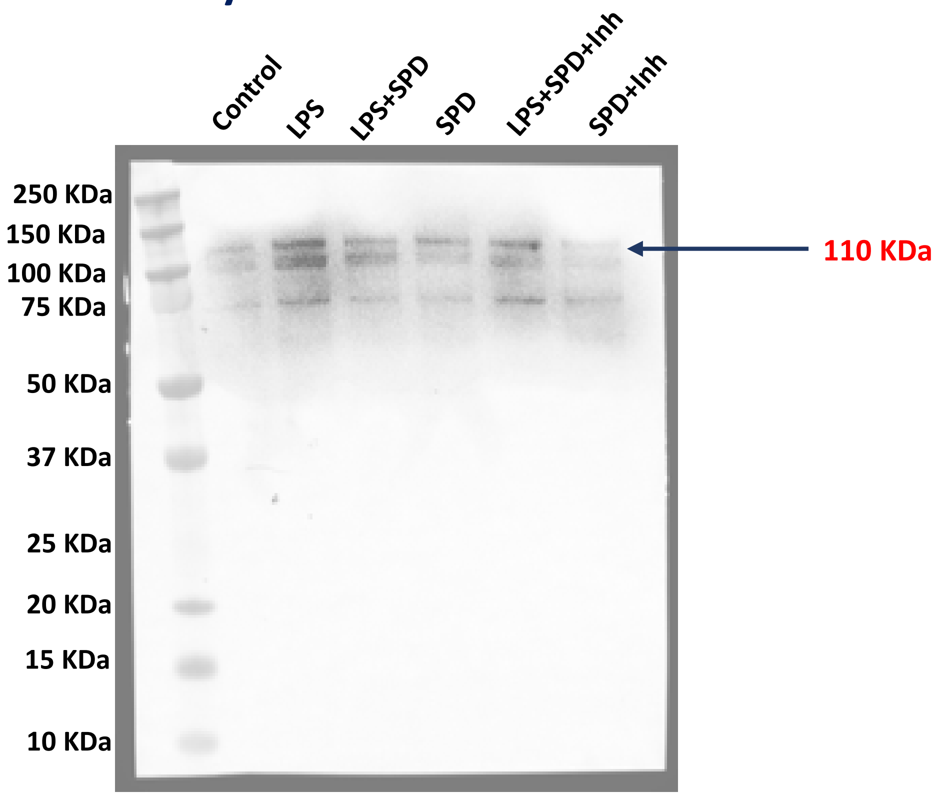

WHOLE WESTERN BLOTS

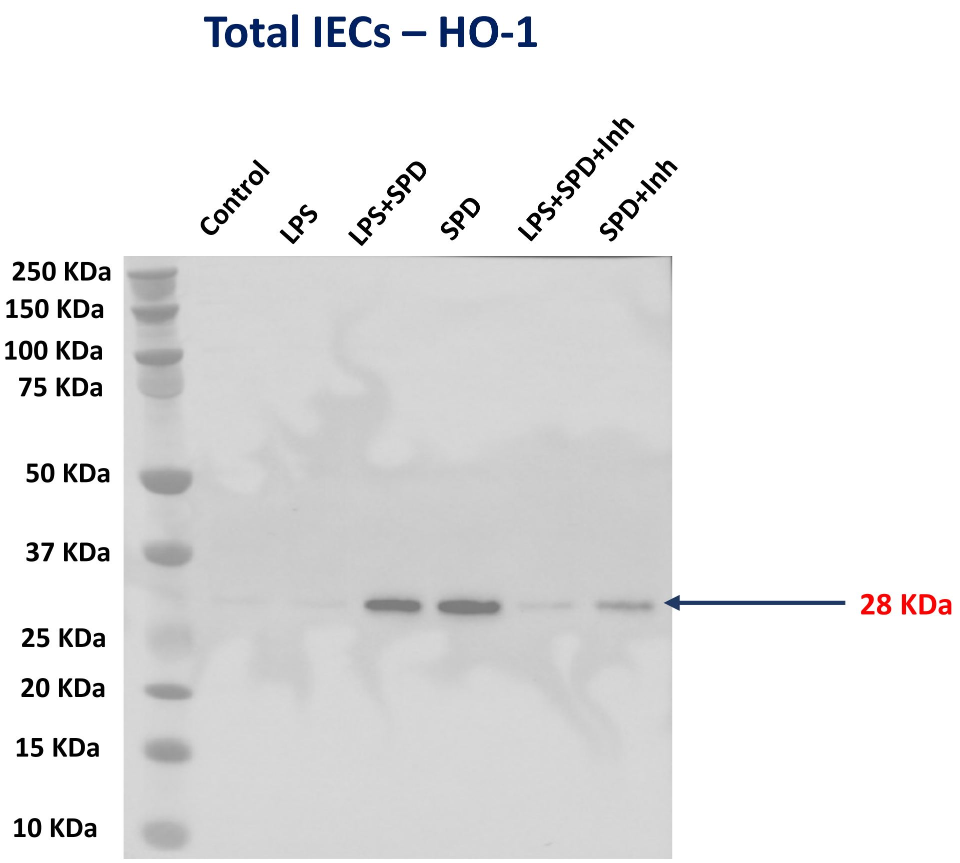

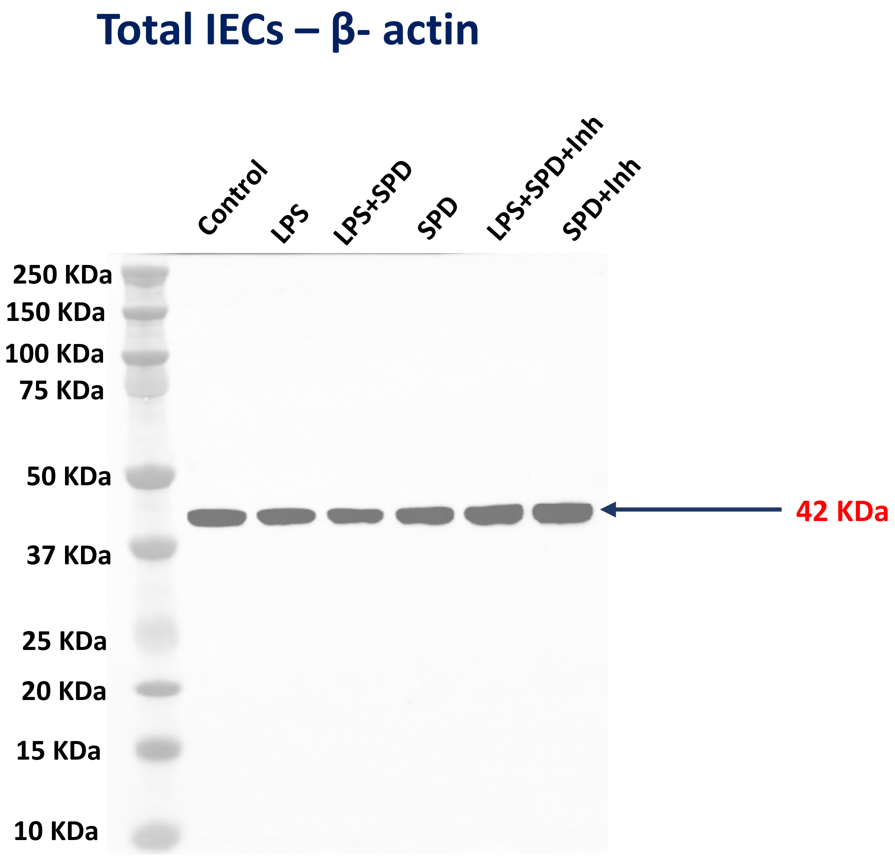

WHOLE WESTERN BLOTS

Small Intestine tissue – AhR

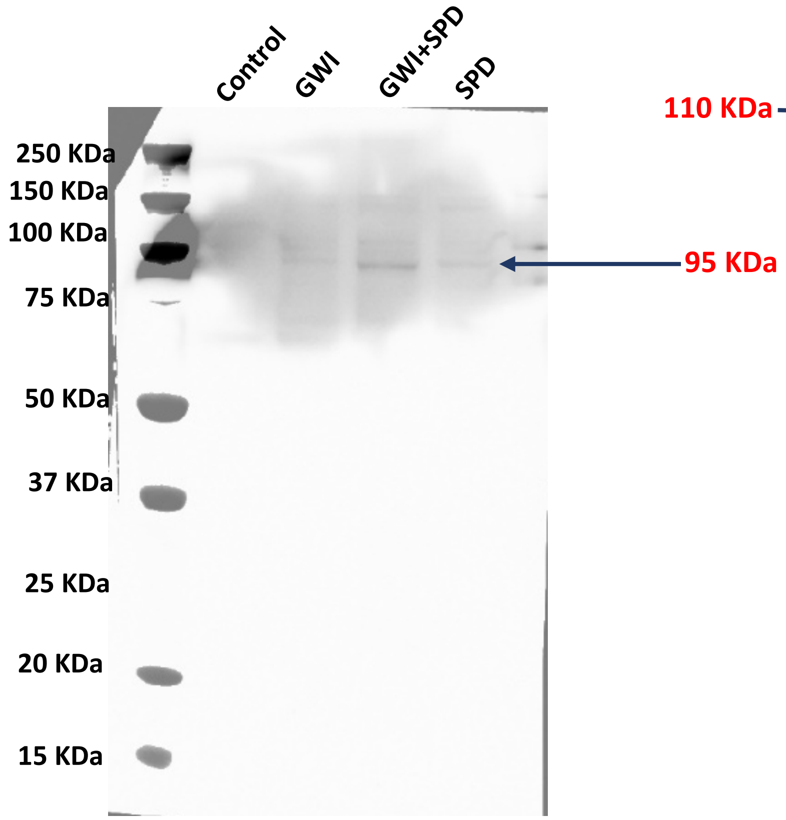

Small Intestine tissue – Nrf2

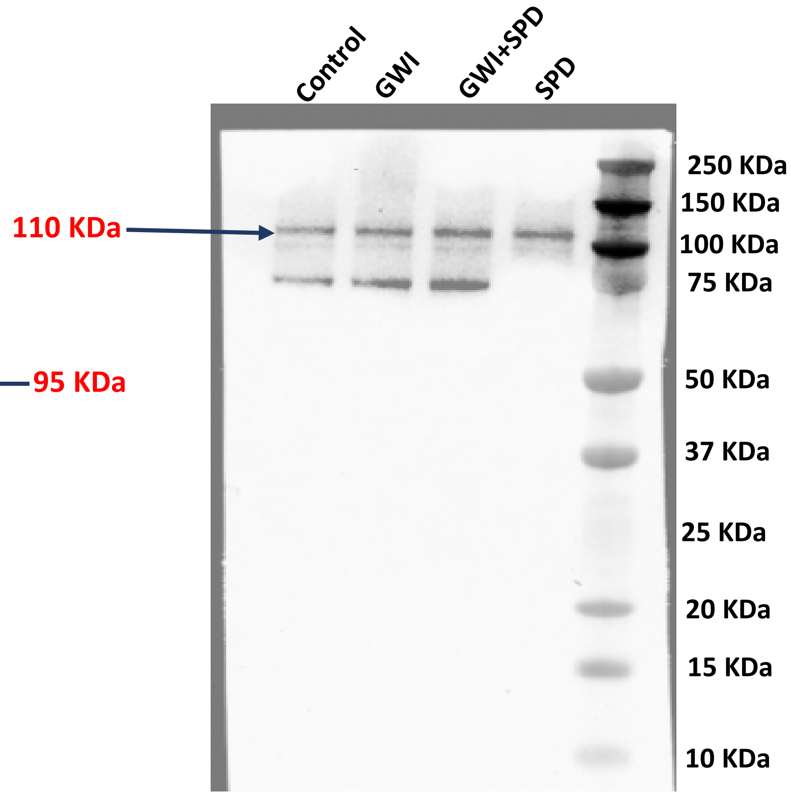

Small Intestine tissue– β- actin

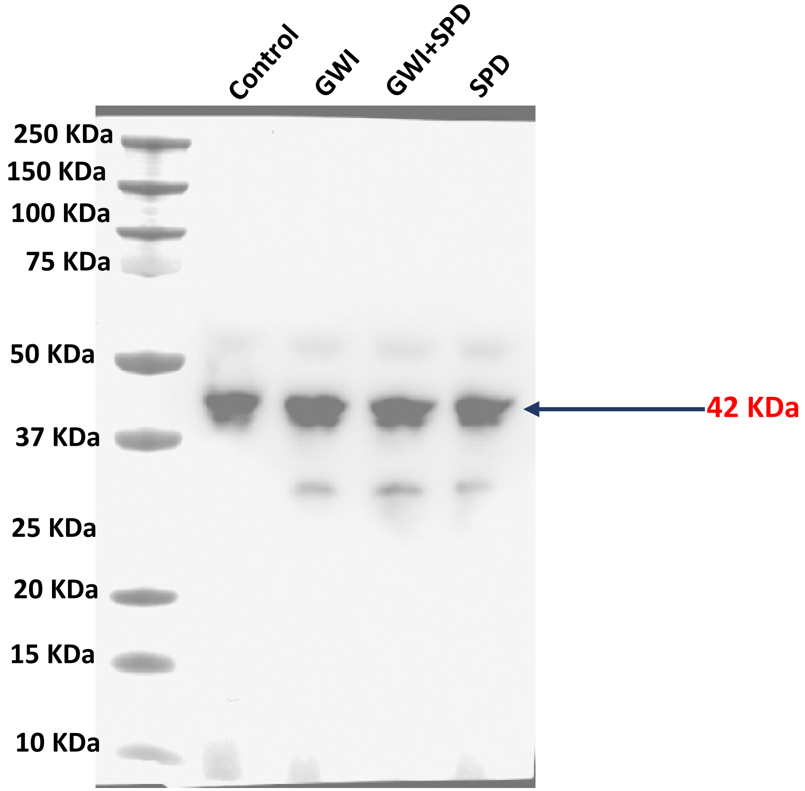

WHOLE WESTERN BLOTS

Small Intestine tissue– HO-1

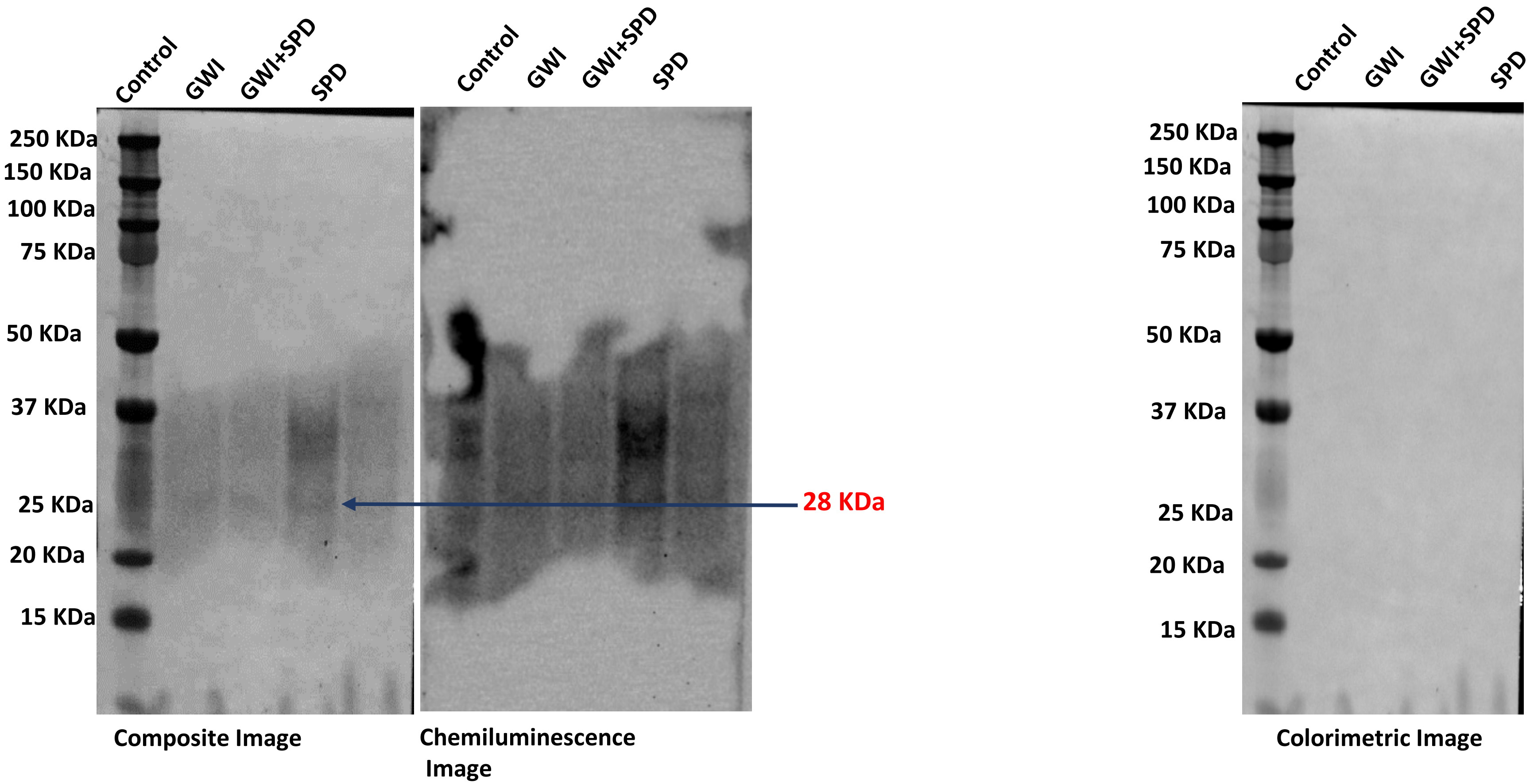

Supplement: Supplementary file 1 — (PDF 1.74 MB) [file 12035_2026_5763_MOESM1_ESM.pdf]
